# Pptc7 is an essential phosphatase for promoting mammalian mitochondrial metabolism and biogenesis

**DOI:** 10.1038/s41467-019-11047-6

**Published:** 2019-07-19

**Authors:** Natalie M. Niemi, Gary M. Wilson, Katherine A. Overmyer, F.-Nora Vögtle, Lisa Myketin, Danielle C. Lohman, Kathryn L. Schueler, Alan D. Attie, Chris Meisinger, Joshua J. Coon, David J. Pagliarini

**Affiliations:** 1 Morgridge Institute for Research, Madison, WI 53715 USA; 2 Department of Biochemistry, University of Wisconsin–Madison, Madison, WI 53706 USA; 3 Department of Chemistry, University of Wisconsin–Madison, Madison, WI 53706 USA; 4 Genome Center of Wisconsin, Madison, WI 53706 USA; 5 Institute of Biochemistry and Molecular Biology, ZBMZ, Faculty of Medicine, University of Freiburg, Freiburg im Breisgau, 79104 Germany; 6 Signalling Research Centres BIOSS and CIBSS, University of Freiburg, Freiburg im Breisgau, 79104 Germany; 7 Department of Biomolecular Chemistry, University of Wisconsin–Madison, Madison, WI 53706 USA

**Keywords:** Metabolomics, Phosphorylation, Energy metabolism, Mitochondrial proteins

## Abstract

Mitochondrial proteins are replete with phosphorylation, yet its functional relevance remains largely unclear. The presence of multiple resident mitochondrial phosphatases, however, suggests that protein dephosphorylation may be broadly important for calibrating mitochondrial activities. To explore this, we deleted the poorly characterized matrix phosphatase *Pptc7* from mice using CRISPR-Cas9 technology. Strikingly, *Pptc7*^*−/*−^ mice exhibit hypoketotic hypoglycemia, elevated acylcarnitines and serum lactate, and die soon after birth. *Pptc7*^*−/−*^ tissues have markedly diminished mitochondrial size and protein content despite normal transcript levels, and aberrantly elevated phosphorylation on select mitochondrial proteins. Among these, we identify the protein translocase complex subunit Timm50 as a putative Pptc7 substrate whose phosphorylation reduces import activity. We further find that phosphorylation within or near the mitochondrial targeting sequences of multiple proteins could disrupt their import rates and matrix processing. Overall, our data define Pptc7 as a protein phosphatase essential for proper mitochondrial function and biogenesis during the extrauterine transition.

## Abstract (web summary)

The mitochondria houses several phosphatases, but their function is not well characterized. Here, the authors show that mitochondrial phosphatase Pptc7 is important during development for proper mitochondrial function and has a role regulating protein import with the translocase subunit Timm50.

## Introduction

Mitochondria are multifaceted organelles required for metabolic and signaling processes within almost every eukaryotic cell type^1^. Beyond their production of ATP through oxidative phosphorylation, mitochondria play key roles in biosynthesis, ion homeostasis, redox signaling, and apoptotic cell death—activities that must be calibrated to changing cellular needs. Recent investigations suggest that these and other mitochondrial functions may be affected by post-translational modifications (PTMs), such as phosphorylation^2,3^ and acylation^4,5^. These PTMs are found on hundreds of mitochondrial proteins^6,7^, and can alter enzyme function^6^, complex assembly^8^, and metabolic flux^9,10^. However, other studies suggest that these mitochondrial PTMs can arise non-enzymatically^11,12^ and are often found at low stoichiometry^2,13–15^, calling into question whether and to what extent these modifications exert regulatory functions.

Although much remains to be established about the overall nature and importance of reversible phosphorylation in mitochondria, it is clear that these organelles possess a number of resident phosphatases^16–18^. For example, it has long been known that the pyruvate dehydrogenase^9^ and branched-chain ketoacid dehydrogenase complexes^19^ include bound phosphatases (and kinases) that regulate their activities. However, beyond these, there exist other poorly characterized mitochondrial proteins that possess known or predicted protein phosphatase domains^16–18^, suggesting that protein dephosphorylation may be of more widespread importance in mitochondria than is currently appreciated.

To begin exploring the importance of mitochondrial protein dephosphorylation in mammalian systems, we chose to investigate *Pptc7*, a poorly characterized, matrix-localized PP2C phosphatase^17^. Numerous lines of evidence suggest that Pptc7 may play a key role in supporting mammalian mitochondrial metabolism. First, the *Saccharomyces cerevisiae* ortholog of *Pptc7*, *PTC7*, is regulated in response to nutrient switching^20^, and prior investigations have connected both the yeast and human homologs to the support of mitochondrial functions^17,18,21,22^. Further, *Pptc7* expression is diminished in liver tissue from obese (*ob/ob*) C57BL/6 mice^23^, which have altered mitochondrial phosphorylation profiles and metabolic dysfunction^6^. Finally, *Pptc7* expression is circadian in mouse liver^24^, with cyclic expression consistent with the metabolic switches between the fasted and fed states. Collectively, these data led us to hypothesize that Pptc7 may dephosphorylate mitochondrial proteins to help enable metabolic transitions in mammals.

To test this, we used CRISPR-Cas9 to generate a global *Pptc7* knockout in *Mus musculus*. We find that *Pptc7*^*−/−*^ mice are born at the expected Mendelian frequency, but exhibit severe metabolic phenotypes, including hypoketotic hypoglycemia, and die within one day of their birth. Proteomic and phosphoproteomic analyses suggest that Pptc7 may influence numerous mitochondrial processes, and that its loss causes post-transcriptional downregulation of mitochondrial content and disrupts key metabolic shifts required for a successful extrauterine transition. Furthermore, our data suggest that Pptc7 influences mitochondrial protein import and processing via two mechanisms: through dephosphorylation of the mitochondrial import complex protein Timm50, and via dephosphorylation of residues within or proximal to the targeting sequences of various imported proteins. Collectively, our data argue that proper management of mitochondrial protein phosphorylation is of vital importance to mammalian metabolism and development.

## Results

### Global knockout of *Pptc7* causes perinatal lethality

**Fig. 1:**
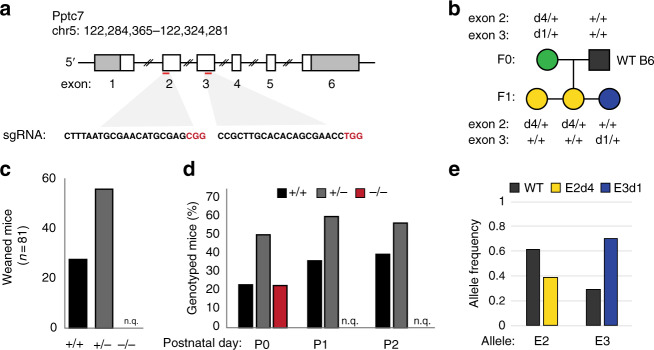
Global, CRISPR-mediated knockout of *Pptc7* causes perinatal lethality. **a** Targeting strategy for the *Pptc7* locus in *Mus musculus* is shown; two sgRNAs were designed to exons 2 and 3 and injected simultaneously into a one cell zygote. **b** The founder mouse (green circle) was bred to a wild type C57B6/J mouse (gray square), generating F1 pups possessing deletions (labeled dX where X is number of base pairs deleted) in exon 2 (yellow circles) or exon 3 (blue circles). This segregation pattern indicates the founder mouse is compound heterozygous (null) for *Pptc7*. **c** No *Pptc7* knockout pups were found at weaning (*n* = 81; n.q.—not quantified). **d** *Pptc7* knockout pups are born at Mendelian frequencies, but no live pups were found at postnatal day 1 (P1) (*n* = 14) or day 2 (P2) (*n* = 17) (reported as n.q.—not quantified). **e** Frequency of WT (gray) or mutant (yellow, E2; blue, E3) alleles at exon 2 (E2) and exon 3 (E3) in skeletal muscle of the F0 founder mouse, demonstrating mosaicism

*Pptc7* is a PP2C phosphatase that has been localized to mitochondria via organellar proteomics^16^ and immunofluorescence^25^. APEX-tagging and subfractionation approaches have further localized human PPTC7 and its yeast ortholog Ptc7p to the matrix compartment^26,27^. To understand the role of Pptc7 in mammalian mitochondria, we generated a CRISPR-mediated global knockout model in *Mus musculus*. Exons 2 (E2) and 3 (E3) were targeted simultaneously (Fig. 1a), generating a founder mouse carrying indels in each region (Supplementary Fig. 1a). Each indel caused a frameshift that negated key catalytic residues and truncated the protein, and thus generated bona fide null alleles (Supplementary Fig. 1b). Upon breeding, the founder produced F1 progeny each carrying only one of the indels, thereby generating two lines carrying distinct Pptc7-null alleles (Fig. 1b). This inheritance pattern indicated that the founder mouse was compound heterozygous (null) for *Pptc7*, yet this mouse showed no overt phenotypes for ~18 months.

We interbred *Pptc7*^*+/−*^ mice from both lines and genotyped their progeny, but found no *Pptc7*^*−/−*^ (KO) mice in the weaned pups (*n* = 130). This observation suggests that CRISPR-mediated loss of *Pptc7* via either indel causes lethality that is not due to off-target effects (Fig. 1c, Supplementary Figs. 1c–e). We found that pups of all genotypes are born at Mendelian frequencies (Fig. 1d, Supplementary Figs. 1f, h), but fail to survive after the perinatal transition (Fig. 1d).

The full penetrance of perinatal lethality in the *Pptc7* knockout pups led us to investigate how the founder mouse, who was compound heterozygous for *Pptc7*, survived. CRISPR-generated mice are often mosaic^28^, suggesting that the founder may have expressed a non-Mendelian ratio of wild type and null alleles in its somatic tissues. To test this, we performed next generation sequencing (Supplementary Fig. i) and found skewed allele frequencies in all tissues examined from the founder (Supplementary Figs. 1j–p), while F1 progeny had expected allele frequencies (Supplementary Figs. 1q–t). Notably, the founder had a substantial percentage of wild type alleles (Fig. 1e), which likely contributed to survival. Collectively, these data demonstrate that *Pptc7* expression is required for survival of the extrauterine transition in mice, and that CRISPR-Cas9 associated mosaicism serendipitously enabled survival of the founder mouse and germline transmission of knockout alleles for an essential gene.

### *Pptc7*^*−/−*^ mice have defects similar to inborn errors of metabolism

**Fig. 2:**
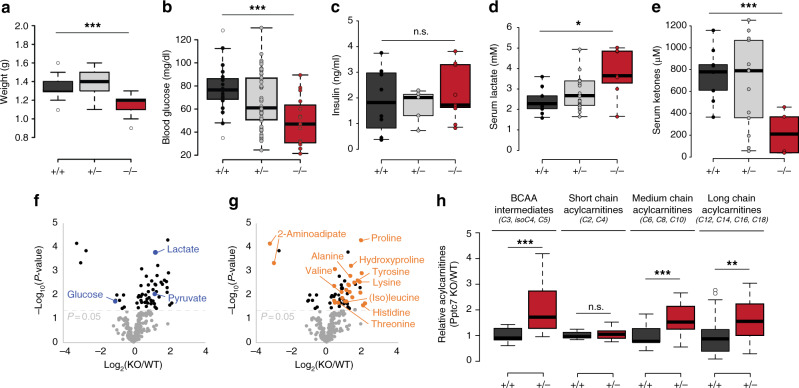
*Pptc7*-null mice have defects associated with inborn errors of metabolism. **a** *Pptc7* KO perinatal pups (P0, red, *n* = 24) weigh significantly less than wild type (dark gray, *n* = 25) and heterozygous (light gray, *n* = 60) littermates. **b** *Pptc7* KO pups (*n* = 15) are hypoglycemic relative to WT (*n* = 19) and heterozygous (*n* = 52) littermates **c** *Pptc7* KO pups (*n* = 10) show no difference in circulating insulin relative to WT (*n* = 8) and heterozygous (*n* = 5) littermates. **d** *Pptc7* KO pups (*n* = 7) have elevated serum lactate relative to WT (*n* = 9) and heterozygous (*n* = 22) littermates. **e** *Pptc7* KO pups (*n* = 6) have lower concentrations of serum ketones relative to WT (*n* = 9) and heterozygous (*n* = 13) pups. **f** Metabolomics data from liver tissues reveal decreased glucose and increased lactate and pyruvate in KO (*n* = 5) samples relative to WT (*n* = 5). **g** Metabolomics data from liver tissue (same data as shown in  **f**) reveal numerous differences in amino acids and their intermediates in KO samples relative to WT. **h** Acylcarnitine analysis of liver tissues from KO and WT. For each plot, *n* = 7 tissues per genotype for each reported acylcarnitine species. For box plots in **a**–**e** and **h**, center lines show the medians; box limits indicate the 25th and 75th percentiles as determined by R software; whiskers extend 1.5 times the interquartile range from the 25th to 75th percentiles, and outliers are represented by white dots. Significance calculated by a two-tailed Student’s *t*-test; * = *p* < 0.05, ** = *p* < 0.01, *** = *p* < 0.001, n.s. = not significant (*p* > 0.05). Source data for panels **f**, **g**, and **h** are provided as a Source Data file

During birth, mammals transition from a primarily glycolytic metabolism *in utero* to a reliance on lipid- and protein-rich milk as a nutrient source^29,30^. As such, deficiency in metabolic processes including glycogen mobilization^31^, fatty acid oxidation^32^, and ketone body utilization^33^ can lead to perinatal lethality. We profiled these and other metabolic phenotypes to assess their potential contribution to the early *Pptc7*^*−/−*^ lethality. *Pptc7* KO pups (Fig. 2a, Supplementary Fig. 2a–c) and E14.5 embryos (Supplementary Fig. 2d) weighed significantly less than their wild type (WT) counterparts, suggesting metabolic deficiencies both in utero and after birth. *Pptc7* KO pups were also hypoglycemic (Fig. 2b), with a median blood glucose of 47 mg/dl relative to 77 mg/dl for WT pups. Insulin levels were unchanged in KO relative to WT pups (Fig. 2c), suggesting that their hypoglycemia may alternatively have arisen through impaired gluconeogenesis and/or increased glycolytic flux. Consistently, KO pups displayed serum lactate levels over 5 mM (Fig. 2d), which is commonly associated with lactic acidosis and mitochondrial dysfunction^34^. Finally, KO mice were hypoketotic, with a ~3.5-fold decrease in serum ketones relative to their WT littermates (Fig. 2e), and analysis of matched glucose and ketone levels across mice demonstrates hypoketotic hypoglycemia (Supplementary Fig. 2e).

Hypoketotic hypoglycemia is typically associated with defects in fatty acid oxidation (FAO)^35^, but can also occur with more generalized mitochondrial defects, such as mtDNA loss^36^. To further explore the underlying cause of this phenotype, we examined metabolites and acylcarnitines in tissues isolated from newborn WT and KO pups. Consistent with the low blood sugar and elevated lactic acid seen in knockout serum, metabolomic analysis of liver tissue revealed decreased glucose levels concomitant with increased pyruvate and lactate levels (Fig. 2f), suggesting a defect in gluconeogenesis. This analysis also revealed a substantial increase in amino acids whose catabolism occurs within mitochondria, such as branched-chain amino acids (BCAAs), in both liver (Fig. 2g) and heart (Supplementary Fig. 2f). These data were corroborated via acylcarnitine analysis, as byproducts of BCAA catabolism were all elevated in *Pptc7* knockout liver (Fig. 2h, Supplementary Fig. 2h) and heart (Supplementary Fig. 2g, i). These acylcarnitine data also suggest widespread defects in FAO, as medium and long-chain acylcarnitines were significantly increased in *Pptc7* KO liver and heart tissues, and short chain acylcarnitines levels were increased in heart (Fig. 2h, Supplementary Fig. 2g, i).

To our knowledge, these *Pptc7* KO-associated metabolic abnormalities are not consistent with any single inborn error of metabolism, but rather share molecular characteristics with various disorders. For instance, glutaric aciduria type II (GAII) has substantial overlap with *Pptc7* KO presentation (hypoketotic hypoglycemia, increased circulating lactate, and elevation of multiple acylcarnitines)^37^, yet key distinctions remain (e.g., levels of α-aminoadipic acid, a lysine catabolite, are typically increased in GAII instead of decreased, as is seen in the *Pptc7* KO)^37^. In this way, the pleiotropic effects of Pptc7 disruption on FAO might be most analogous to the established paradigm of synergistic heterozygosity, in which combined heterozygous defects in distinct FAO enzymes is sufficient to produce a metabolic phenotype^38^. Overall, these data suggest that Pptc7 function likely influences multiple metabolic pathways, and that its expression is required for the use of multiple nutrients in the perinatal stage.

### *Pptc7*^*−/−*^ tissues have defects in mitochondrial biogenesis

**Fig. 3:**
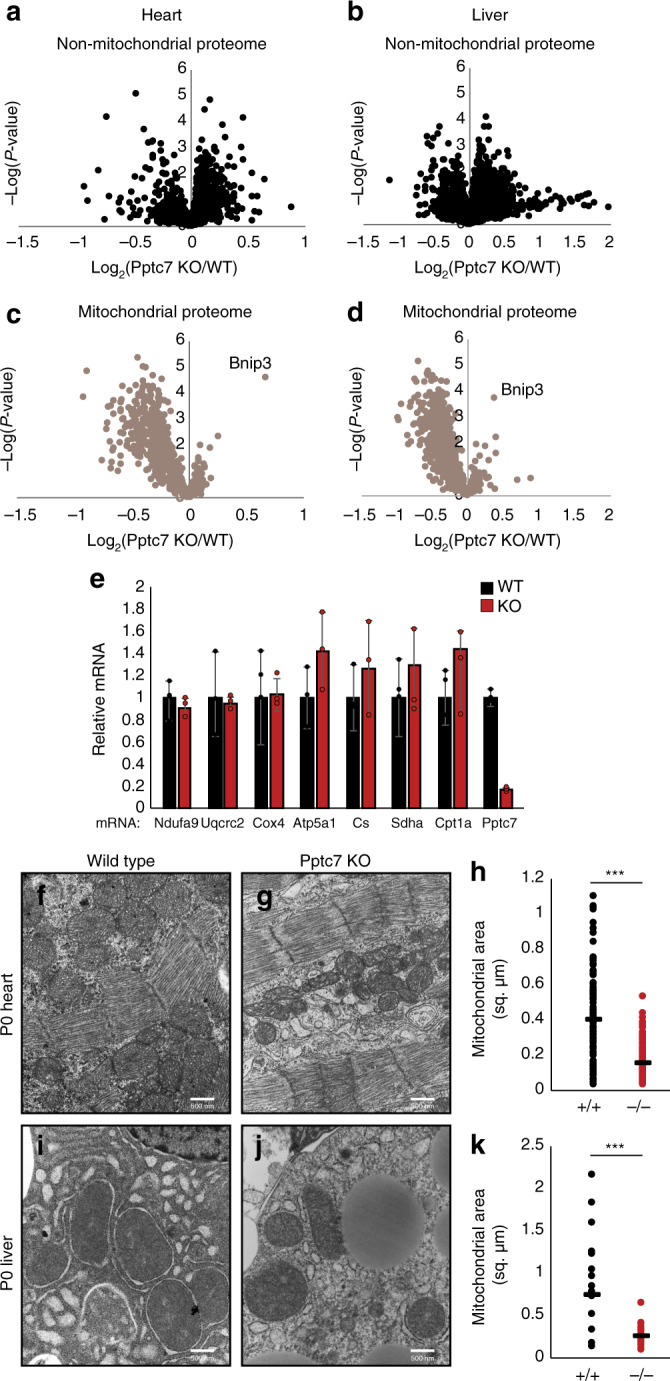
Loss of Pptc7 selectively decreases mitochondrial content. **a**–**d** Volcano plots of non-mitochondrial (**a**, **b**) proteins and mitochondrial (**c**, **d**) proteins in both heart (**a**, **c**) and liver (**b**, **d**); *n* = 5 tissues per genotype. One of the only significantly increased mitochondrial proteins is Bnip3, a protein induced during organellar stress. **e** qPCR of select nuclear-encoded mitochondrial targets from WT (black, *n* = 4) or KO (red, *n* = 3) heart tissue shows no significant decrease (*p* > 0.05) between genotypes, except for *Pptc7*. Error bars represent standard deviation; significance calculated with a two-tailed Student’s *t*-test. **f**–**h** Transmission electron microscopy (TEM) was used to image heart tissue from WT (**f**) and *Pptc7* KO (**g**) mice. Mitochondrial area was quantified in ImageJ with size extrapolated from the scale bar (500 nm). KO heart mitochondria (*n* = 110) are significantly smaller than those in WT tissues (*n* = 105) (**h**). **i**–**k** TEM images of liver tissue from WT (**i**) and *Pptc7* KO (**j**) and analyses as described for heart tissue; KO liver mitochondria (*n* = 17) are significantly smaller than WT mitochondria (*n* = 20) (**k**). For **h**, **k**, each dot represents the area of a single quantified mitochondrion in WT (black) or KO (red) tissues; the line represents the median area in each condition. For statistical analysis, significance was calculated using a two-tailed Student’s *t*-test; *** = *p* < 0.001. Source data for panels **a**–**d** are provided as Supplementary Datasets 1 and 2

To profile the molecular consequences of Pptc7 loss, we performed quantitative proteomics on tissues from WT and KO littermates. These results showed disruption of non-mitochondrial proteins in KO heart (Fig. 3a, Supplementary Dataset 1) and liver (Fig. 3b, Supplementary Dataset 2), with gene enrichment signatures suggesting dysregulated RNA splicing, nuclear import, glutamine metabolism, and protein translation (Supplementary Fig. 3a, b). Most strikingly, KO tissues also displayed a widespread decrease of mitochondrial proteins in both tissues (Fig. 3c, d, Supplementary Fig. 2c, g). These data were corroborated by western blots, which showed that proteins involved in oxidative phosphorylation were only decreased in the KO mice (Supplementary Fig. 3d, h). Notably, Bnip3—a stress-activated protein involved in mitophagy^39^—is elevated in KO tissue, suggesting that mitophagy might play a role in these decreased protein levels. Importantly, nuclear-encoded mitochondrial mRNA transcripts were not decreased (other than the CRISPR-targeted *Pptc7*) (Fig. 3e), suggesting that disruption of mitochondrial protein homeostasis in *Pptc7*-null tissues occurs post-transcriptionally.

To further examine how this loss of mitochondrial proteins is manifest at the cellular level, we performed electron microscopy of tissue slices from P0 mice. In both heart (Fig. 3f, g) and liver (Fig. 3i, j) mitochondria from KO mice were significantly smaller than those from WT mice. On average, mitochondria from KO mice had ~40% (Fig. 3h) and ~34% (Fig. 3k) of the surface area of WT mice in heart and liver, respectively. As such, certain mitochondrial defects that we observe in KO tissues, such as decreased citrate synthase activity (Supplementary Fig. 3e, i) and coenzyme Q levels (Supplementary Fig. 3f, j), likely stem from the overall reduction in mitochondrial content, and not from protein-level regulation; in each case, these changes are no longer apparent when normalized to total mitochondrial protein content (Supplementary Fig. 3e, f, i, j). To determine putative Pptc7 substrates whose dysregulation might contribute to these global mitochondrial effects, we next performed phosphoproteomic analysis of WT and KO tissues.

### Phosphoproteomic analysis of *Pptc7*^*−/−*^ mice reveals candidate substrates

**Fig. 4:**
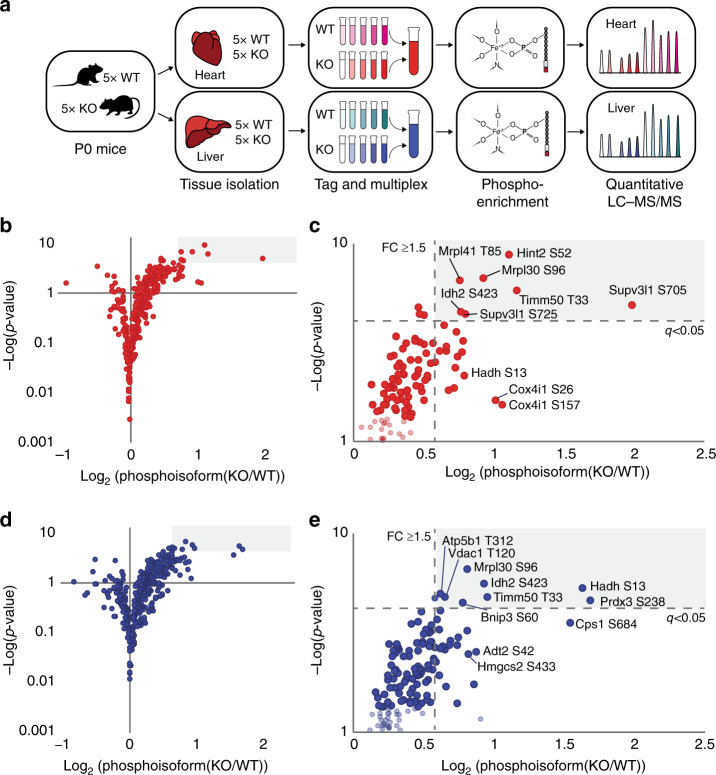
Phosphoproteomic analyses reveal candidate Pptc7 substrates. **a** Schematic of multiplexed, quantitative phosphoproteomics for *Pptc7* WT and KO heart (red) and liver (blue) tissues (*n* = 5 for each genotype in each tissue). **b** Volcano plot showing mitochondrial phosphoisoforms as Log_2_(fold change) in *Pptc7* KO versus WT heart. Shaded region corresponds to identified events with ≥ 1.5-fold change and a Benjamini–Hochberg multiple hypothesis-adjusted *q*-value of < 0.05. **c** Zoom in of shaded region in **b** showing select statistically significant phosphoisoforms that are candidate Pptc7 substrates. **d** Volcano plot showing mitochondrial phosphoisoforms as Log_2_(fold change) in *Pptc7* KO versus WT liver. Shaded region corresponds to identified events with ≥ 1.5-fold change and a Benjamini–Hochberg multiple hypothesis-adjusted *q*-value of < 0.05. **e** Zoom in of shaded region in **d** showing select statistically significant phosphoisoforms that are candidate Pptc7 substrates. Source data for panels **b**–**e**. are provided as Supplementary Datasets 1 and 2

We previously demonstrated that deletion of the *S. cerevisiae* ortholog of Pptc7, Ptc7p, caused mitochondrial dysfunction concomitant with elevated phosphorylation on a range of proteins^17^. These results suggest that this phosphatase likely has multiple substrates. We similarly assessed putative substrates for Pptc7 using a quantitative multiplexed phosphoproteomic analysis of littermate-matched WT and KO heart and liver tissues (Fig. 4a, Supplementary Datasets 1–2). Analogous to ∆*ptc7* yeast, we find that 28% of detected mitochondrial phosphorylation events are altered between WT and KO tissues, compared to only 4% of non-mitochondrial phosphoisoforms (Supplementary Fig. 4a). Of mitochondrial phosphoisoforms changing by ≥ 1.5-fold, 98% are elevated (Supplementary Fig. 4b), consistent with the expected outcome of disrupting a mitochondria-specific phosphatase. In heart, 21 mitochondrial phosphoisoforms were elevated by ≥ 1.5-fold with a *p*-value of < 0.05 (two-tailed Student’s *t*-test, Fig. 4b, Table 1), seven of which had a Benjamini–Hochberg multiple hypothesis-adjusted *q*-value < 0.05 (Fig. 4c). Similarly, liver exhibited 28 (Fig. 4d, Table 1), and eight (Fig. 4e) phosphoisoforms meeting these same respective criteria.

**Table 1:**
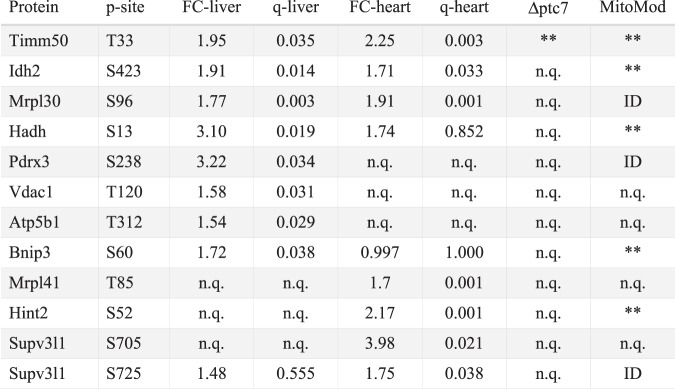
Select Pptc7 candidate substrates across liver, heart, and knockout yeast

Groups of phosphoisoforms shown by protein name and phosphosite identified (labeled p-site in table), with relative fold changes (FC) and *q*-values (calculated by Benjamini–Hochberg multiple hypothesis correction, labeled q in table) in heart and liver *Pptc7* KO tissues. Identification of phosphoproteins in Δ*ptc7* yeast^17^ and in our previous study of obese mice (MitoMod)^6^ also reported; ** = significantly changing as calculated by a two-tailed Student’s *t*-test for the Δ*ptc7* dataset or by multiple hypothesis testing via FDR calculation as detailed in ref. ^6^. ID = identified; n.q. = not quantified. Timm50 (top) was significantly altered in both tissues and both previous studies, warranting biological follow up

Mitochondrial phosphoisoforms elevated in *Pptc7* KO tissues spanned numerous pathways (Table 1, Supplementary Datasets 1–2), including mitochondrial RNA processing and translation (Mrpl30, Mrps26, Lrpprc, and Supv3l1) (Supplementary Fig. 4c), the TCA cycle (Idh2, Aco2, and Cs) (Supplementary Fig. 4d), and fatty acid oxidation (Hadh and Etfa) (Supplementary Fig. 4e). While most identified proteins reside within the same matrix compartment as the phosphatase, others, such as Vdac1, may have increased phosphorylation due to indirect effects of Pptc7 ablation. Notably, we identified the overlap between a subset of these elevated phosphoproteins and those found in ∆*ptc7* yeast^17,18^ (Table 1), suggesting that select phosphatase functions are conserved. Further, many potential Pptc7 substrates were identified in a mouse model of obesity and type 2 diabetes^6^ (Table 1). This broad profile of phosphoproteins is consistent with Pptc7 affecting diverse proteins and pathways, and suggests that it may influence multiple processes within mitochondria.

### The Pptc7 substrate Timm50 is conserved through *S. cerevisiae*

**Fig. 5:**
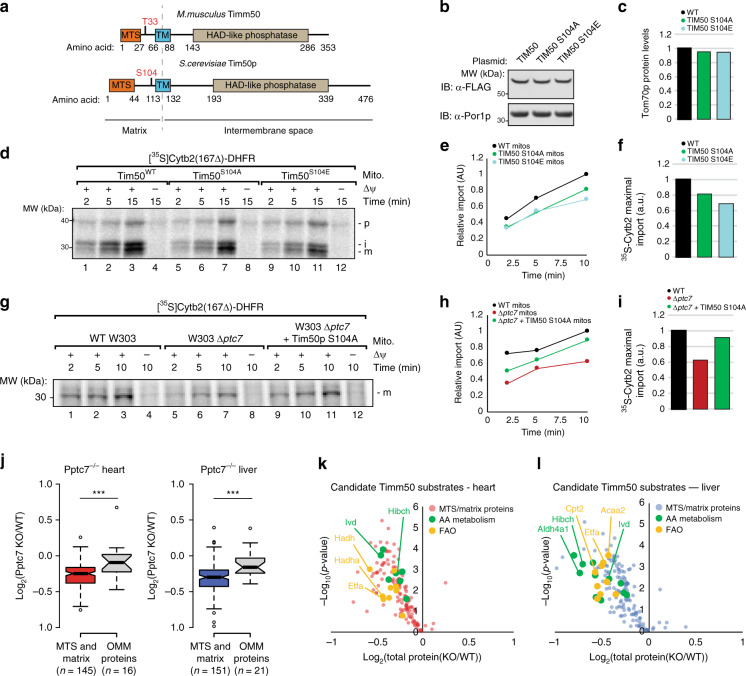
Phosphorylation of Timm50 decreases mitochondrial protein import. **a** Protein domain schematic of mouse Timm50 (top) and yeast Tim50p (bottom) with highlighted phosphoresidues (red). **b** Western blot of overexpressed FLAG-tagged Tim50p WT, S104A, or S104E shows that mutations do not destabilize proteins. **c** Quantification of western blot for Tom70p protein levels (see Supplementary Figure 5F for corresponding western blot data). **d** Import assays in Δ*tim50* yeast mitochondria overexpressing wild type (WT) TIM50 or S104 mutants using generic import cargo cytochrome b_2_-(167)_∆19_-DHFR. Imported proteins result in accumulation of the mature (m) versus precursor (p) or intermediate (i) processed bands. Quantification of import rates is shown over time (**e**) and as maximal import signal (**f**) (AU densitometry at 10 min). **g** Import assays in mitochondria isolated from WT, Δ*ptc7*, or Δ*ptc7* overexpressing TIM50 S104A yeast using the matrix-targeted model substrate cytochrome b_2_-(167)_∆19_-DHFR. Imported proteins result in accumulation of the mature (m) product. Quantification of import rates is shown over time (**h**) and as maximal import signal (**i**) (AU densitometry at 10 min). **j** Quantification of mitochondrial proteins in mouse heart (left) (*n* = 145 for matrix proteins and *n* = 16 for OMM proteins) and mouse liver (right) (*n* = 151 for matrix proteins and *n* = 21 for OMM proteins) showing significantly lower expression (*** = *p* < 0.001, as calculated by a two-tailed Student’s *t*-test) of candidate Timm50 substrates (e.g., MTS and matrix-containing proteins) than proteins that localize to the outer mitochondrial membrane (OMM). For box plots in **j**, center lines show the medians; box limits indicate the 25th and 75th percentiles as determined by R software; whiskers extend 1.5 times the interquartile range from the 25th and 75th percentiles, and outliers are represented by white dots. **k**, **l** Volcano plots of candidate Timm50 substrates in heart (**k**) and liver (**l**) show downregulation of metabolic proteins in amino acid metabolism and fatty acid oxidation, both of which are disrupted in Pptc7-null tissues (Fig. 2). Source data for panels **b**, **d**, **g**, and **j**–**l** are provided as a Source Data file

We next sought to identify candidate Pptc7 substrates from our analyses above whose dysregulation could give rise to the marked mitochondrial dysregulation we observe in the KO mice. Timm50 is among the most significantly elevated phosphoproteins in *Pptc7* KO tissues, and its identification in both *PTC7* knockout yeast^17^ and our previous study of obese mice^6^ (Table 1) suggests that this phosphorylation may have functional relevance under multiple conditions. The phosphorylation site on Timm50 (Tim50p in yeast) occurs on the matrix-facing N-terminal tail in both the yeast and mouse orthologs (Fig. 5a). Importantly, Ptc7p can directly dephosphorylate Tim50p that is site-specifically phosphorylated at S104 (Supplementary Fig. 5a), suggesting this phosphoprotein can be a direct substrate of the phosphatase in yeast. Although *Timm50* is essential in both *S. cerevisiae*^40^ and *M. musculus*^41^, we hypothesize that a less severe disruption of Tim50p/Timm50-mediated protein import activity could generate many of the pleiotropic effects seen in *∆ptc7* yeast and *Pptc7* KO mice. Consistently, a recent study identified compound heterozygous mutations in *TIMM50* in a patient with a mitochondrial disorder whose tissues exhibited a similar decrease in respiratory chain components as the *Pptc7* KO mice^42^.

To test whether Tim50p phosphorylation affects mitochondrial protein import, we first generated *∆tim50* strains harboring a plasmid that encodes FLAG-tagged variants of Tim50p (either WT, a non-phosphorylatable (S104A) mutant, or a phosphomimetic (S104E) mutant). Each version of Tim50p expressed comparably at the protein level (Fig. 5b). These strains showed no detectable growth defects in fermentation or respiratory conditions (Supplementary Figs. 5b–e), and did not differentially express TOM complex components (Fig. 5c, Supplementary Fig. 5f). To test whether Tim50p phosphorylation affects import efficiency, we performed mitochondrial import assays using three independent matrix-bound cargo proteins: Mdh1p, malate dehydrogenase; Atp2p, a subunit of Complex V; and cytochrome b_2_-(167)_∆19_-DHFR—a well-established, matrix-targeted model substrate^43^. Mitochondria isolated from the phosphomimetic Tim50p S104E strain showed import defects with each of these radiolabeled recombinant precursors, with 20–40% decreased import rates in the S104E mutant relative to mitochondria expressing wild type Tim50p (Fig. 5d–f, Supplementary Figs. 5g–j). These defects were reproducible in a second yeast strain in which the endogenous *TIM50* gene is controlled by a galactose-inducible promoter (*GAL7*);^44^ under similar respiratory conditions (in which endogenous TIM50 expression is severely dampened due to the lack of galactose), mitochondria isolated from the S104E strain imported cytochrome b_2_-(167)_∆19_-DHFR almost three-fold less efficiently than WT (Supplementary Figs. 5k, l). Collectively these data suggest that phosphorylation at S104 on Tim50p can dampen the import of multiple matrix-bound proteins into mitochondria.

Given the elevation of Tim50p S104 phosphorylation in Δ*ptc7* yeast^17^, we next tested mitochondrial protein import in Δ*ptc7* yeast and found an approximate three-fold decrease relative to WT import rates (Fig. 5g, Supplementary Fig. 5m, n). Δ*ptc7* yeast do not have decreased levels of Tim50p, nor of any other import machinery component^17^ (Supplementary Fig. 5o), suggesting that Tim50p phosphorylation likely contributes to the decreased import rates. To test this, we overexpressed a non-phosphorylatable Tim50p mutant, S104A, which partially rescued the import defect in Δ*ptc7* yeast (Fig. 5g, quantified in 5h, i). These data suggest that Tim50p phosphorylation at S104 impacts import rates in Δ*ptc7* yeast, but does not account for the full import defect. Notably, multiple components of the import machinery have been identified as candidate Ptc7p substrates^18^, whose phosphorylation may also contribute to the import defects seen in the Δ*ptc7* strain. Collectively, these data suggest that the loss of Ptc7p elevates phosphorylation on Tim50p, amongst other targets, to dampen mitochondrial import in yeast.

Decreased mitochondrial import may help explain the broad decreases in mitochondrial proteins seen in *Pptc7* KO tissues (Fig. 3). Timm50 is a core component of the Timm23 complex that translocates proteins possessing a mitochondrial targeting sequence (MTS) that are typically bound for the matrix^45^. We hypothesized that if Timm50 was selectively impaired in *Pptc7* KO tissues, then matrix-localized MTS-containing proteins would show greater decreases in our proteomics dataset relative to other mitochondrial proteins (e.g., proteins destined for the outer mitochondrial membrane (OMM)). Indeed, proteins that have both an MTS^46^ and matrix localization^26^ were significantly decreased relative to the OMM-localized proteins we measured in both heart and liver (Fig. 5j) KO tissues. These decreased proteins include those whose dysfunction is associated with various dysregulated metabolites we identified in *Pptc7* KO tissues: Aldh4a1 (hyperprolinemia)^47^, Pcca, Pccb, Acad8, and Ivd (BCAA and catabolite acylcarnitine accumulation)^48^, and Etfa and Etfdh (medium and long-chain acylcarnitine accumulation)^35^ (Fig. 5k, l). Together, these data suggest that disruption of Timm50-mediated import could broadly influence metabolic pathways, and may contribute to the widespread defects seen in the *Pptc7* knockout mouse.

### MTS-proximal phosphorylation can influence import and processing

**Fig. 6:**
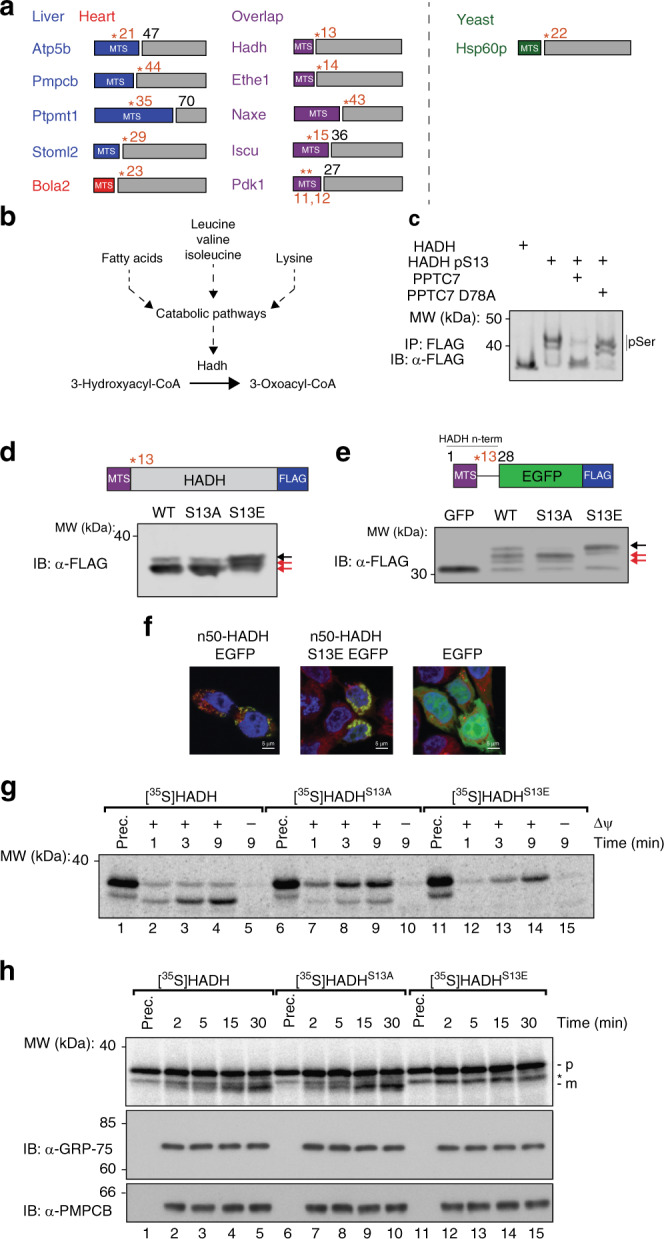
Pptc7 influences import and processing by dephosphorylating MTS residues. **a** Candidate Pptc7 substrates phosphorylated within or proximal to their mitochondrial targeting sequence (MTS) identified in mouse heart (red), mouse liver (blue), both tissues (purple), and Ptc7p-null yeast (green). **b** Hadh is downstream of multiple metabolites dysregulated in Pptc7 knockout mice. **c** Recombinant site-specific incorporated HADH pS13 can be dephosphorylated by recombinant PPTC7, but not a catalytically inactive mutant (D78A). **d** Overexpression of WT, non-phosphorylatable (S13A) and phosphomimetic (S13E) HADH in 293 cells. S13E causes a shift in HADH processing relative to S13A and WT as analyzed by FLAG western blot. **e** A fusion protein of the first 28 amino acids of HADH and GFP-FLAG was made, expressing WT or S13 mutants, as analyzed by FLAG western blot. S13E is sufficient to disrupt processing of the fusion protein. **f** n50-HADH and HADH S13E GFP fusions (containing the first 50 amino acids of HADH, shown in green) overlap with MitoTracker Red staining, unlike EGFP which shows no mitochondrial localization. Nuclei stained in blue (Hoescht stain); scale bar (white) = 5 μm. **g** Import assays with recombinant ^35^S-labeled HADH, S13A, and S13E show that mutation of S13 alters HADH import efficiency into and processing in mitochondria isolated from HEK293T cells. Prec., precursor protein. **h** MPP processing assay shows precursor (labeled “Prec.”) HADH (p, top band) as well as cleavage into the mature form (m, bottom band) for both wild type and S13A HADH. Processing is disrupted in the S13E mutant, shown via the absence of the lowest band. * is a non-specific band in all three conditions. GRP75 and PMPCB western blots demonstrate equal loading. Source data for panels **c**–**e** and **g**–**h** are provided as a Source Data file

Close examination of our phosphoproteomics datasets revealed a second trend related to mitochondrial protein import: amongst elevated (*p* < 0.05, two-tailed Student’s *t*-test) mitochondrial phosphoisoforms identified in mouse and yeast, 11 candidate Pptc7/Ptc7p substrates have phosphorylated residues that lie within or directly proximal to their mitochondrial targeting sequences (Fig. 6a). The localization of these phosphorylation events, coupled with data suggesting that Ptc7p/Pptc7 modulates mitochondrial protein import (Fig. 5), suggest that the phosphatase may play an additional role in import through the dephosphorylation of these “phospho-MTS” substrates.

One such phospho-MTS substrate, Hadh, assists in the metabolism of multiple nutrients that are disrupted in *Pptc7* KO mice (e.g., BCAAs, lysine, fatty acids)^49^ (Fig. 6b), and thus its dysfunction could contribute to the KO metabolic phenotypes. Hadh phosphorylation is also elevated in both tissues, and ranks third in fold change and *q*-value amongst > 9000 phosphoisoforms identified in liver (Fig. 4e, Table 1). To test whether PPTC7 can directly dephosphorylate phospho-MTS substrates, we generated recombinant human HADH with site-specific phosphoserine incorporation at S13^50,51^ (see “Methods” section for details). We validated phosphoserine incorporation using PhosTag gels, which showed a mobility shift for phosphorylated, but not WT, HADH (Fig. 6c, lanes 1 and 2). Further, WT PPTC7, but not an inactive mutant, was able to dephosphorylate HADH (Fig. 6c), as well as the additional phospho-MTS substrates, ETHE1, Naxe, Pdk1, and Iscu (Supplementary Fig. 6a–d).

We next tested whether HADH S13 phosphorylation affects enzyme activity, but found no differences between pS13 and WT HADH (Supplementary Fig. 6e). We reasoned that the proximity of pS13 to the mitochondrial targeting sequence of Hadh may instead affect protein import or processing. Consistently, our proteomics analysis revealed an increased abundance of peptides unique to precursor Hadh (i.e., peptides that span the MTS cleavage site), but not of peptides from the mature (i.e., processed) protein (Supplementary Fig. 6f). To further examine HADH import or post-import protein stability in cells, we expressed phosphomimetic (S13E) and non-phosphorylatable (S13A) HADH in 293 cells and found that the S13E mutant was not properly processed (Fig. 6d). We observed similar protein processing and/or stability errors with phosphomimetic mutation of other phospho-MTS substrates, including ETHE1 (Supplementary Fig. 6g), Naxe (Supplementary Fig. 6h), and Iscu (Supplementary Fig. 6i). To determine whether the MTS-proximal phosphomimetic mutation is sufficient to disrupt processing, we generated chimeras containing the N-terminus of HADH fused to GFP and found that the S13E mutation likewise disrupted processing of the fusion protein (Fig. 6e, Supplementary Fig. 6j). Importantly, the n50-HADH-S13E-GFP chimera still localized to mitochondria (Fig. 6f, Supplementary Fig. 6k), suggesting that the processing defect is not due to altered subcellular targeting.

We next aimed to measure in vitro mitochondrial import efficiency of phospho-MTS substrates. Proteins produced by our in vitro site-specific phospho-incorporation system noted above could not be imported—an established problem for proteins produced in select cell-free and *E. coli*-based systems^52–54^. Instead, we performed import assays with recombinant radiolabeled WT, S13A, and S13E HADH. Each construct could be imported into mitochondria, as evidenced by the time-dependent accumulation of proteinase K-protected species (Fig. 6g); however, HADH S13E was imported more slowly relative to the WT and S13A HADH proteins. Additionally, phosphomimetic mutation of S13 HADH disrupted processing, as only full-length protein accumulated in mitochondria (Fig. 6g). To confirm the processing defect, we subjected HADH, S13A, and S13E mutants to an in vitro MPP processing assay that is uncoupled from protein import^55^. MPP (mitochondrial processing peptidase) removes MTS sequences upon entry into the matrix, and this processing event is critical for mature protein stability^56^. We find that phosphomimetic mutation of S13 completely disrupted HADH processing (Fig. 6h, bottom band), whereas WT and S13A mutants were processed normally. With the caveat that we are analyzing phosphomimetic mutants, these data suggest that phosphorylation of HADH proximal to its MTS is sufficient to dampen, but not eliminate, mitochondrial import, and that dephosphorylation of HADH after translocation is required for MPP processing and mature protein stability. As failure to remove mitochondrial targeting sequences is associated with disrupted protein stability, these data suggest that an inability to dephosphorylate and subsequently process these proteins may further contribute to the decreased mitochondrial content and metabolic defects seen in *Pptc7* KO mice.

## Discussion

Despite numerous studies indicating that many mitochondrial proteins are phosphorylated, how these modifications affect organellar function remains unclear. In the present study, we extended our findings on the yeast mitochondrial phosphatase Ptc7p into a mammalian system through the creation and characterization of a *Pptc7* knockout mouse. Surprisingly, we find that *Pptc7* is an essential gene in mammalian development, with its knockout leading to fully penetrant perinatal lethality.

Perinatal lethality in mouse models is often associated with defects in metabolism, as the newborn pup experiences a robust metabolic transition from placental to extrauterine nutrients^29^. *Pptc7* KO pups present with hypoketotic hypoglycemia—typically associated with FAO disorders^35^—which is the likely cause of their death. Despite this presentation, our molecular characterization using metabolomics, proteomics, and electron microscopy suggests a broader mitochondrial dysfunction, which, in certain circumstances, is also associated with hypoketotic hypoglycemia^36^. For example, knockout of *Cluh*, an RNA-binding protein that selectively influences mitochondrial biogenesis, causes perinatal lethality in mice with metabolic defects (e.g. hypoglycemia and hyperprolinemia) coupled with a stark decrease in the mitochondrial proteome^57^. These data suggest that disruption of genes required for general mitochondrial homeostasis (e.g., *Cluh* and *Pptc7*) can cause symptoms similar to classes of inborn errors of metabolism through widespread mitochondrial dysfunction. Consistently, there are significantly fewer missense mutations in the *PPTC7* gene in the adult human population than predicted by chance^58^, suggesting that its disruption is not well tolerated and is likely selected against in humans.

The global metabolic and mitochondrial defects seen in *Pptc7* KO mice are likely due to aberrantly elevated phosphorylation of numerous mitochondrial proteins. This is similar to our findings in yeast, in which loss of *PTC7* increases phosphorylation on a variety of targets^17,18^. These include multiple components of the import machinery, including Tim50p, Tim44p, Pam16p, and Mas1p, which are localized within the mitochondrial matrix (proximal to the phosphatase), and are essential for the import (Tim50p, Tim44p, Pam16p) and processing (Mas1p) of matrix-bound proteins. Notably, the phosphorylation site found on Tim44p at S168 lies within a key regulatory motif required for interaction with Tim23p and Hsp70p^43,59^, suggesting that Ptc7p-mediated dephosphorylation may affect dynamic protein interactions within the mitochondrial import complex. How Ptc7p affects phosphorylation on these targets will be an important direction for fully understanding its contribution to mitochondrial protein import in *S. cerevisiae*.

In this study, we begin to characterize the effects of Ptc7p on the import of matrix-bound proteins through examination of Tim50p phosphorylation, prioritizing this substrate due to its identification in both Δ*ptc7* yeast and *Pptc7* KO mice. We found that phosphomimetic mutation of S104 on Tim50p decreases the import rates of multiple substrates by 25–40% (Fig. 5). While somewhat modest, this defect would likely cause global decreases in mitochondrial protein content when manifest across multiple matrix-bound targets. Notably, *Pptc7* KO tissues have an ~20% decrease in mitochondrial protein content (Supplementary Figs. 3a, b), including candidate Timm50 substrates, such as fatty acid oxidation enzymes and proteins involved in amino acid catabolism (Fig. 5h–j). These data suggest a model in which phosphorylation-mediated decreases in Timm50 function can cause widespread and pleiotropic effects in mitochondria. Importantly, these data are consistent with a recent report suggesting that compound heterozygous mutation of Timm50 causes lactic acidosis and alters respiratory complex levels in humans^42^—phenotypes similar to those in the *Pptc7* KO mouse.

The notion that the phosphorylation of Tim50p/Timm50 can affect import rates is consistent with previous reports that the mitochondrial import machinery is regulated by kinases in yeast^60–63^. While phosphorylation of TOM complex proteins is plausible given their accessibility to cytoplasmic kinases, less is known regarding phosphorylation on proteins within the TIM complex. Further, how the matrix-localized tail of Timm50—a protein spanning the mitochondrial inner membrane—could be accessed by such regulatory proteins is unknown. While some studies suggest protein kinases (e.g., PKA) lie within the mitochondrial matrix^64,65^, it is not well understood how and when these enzymes translocate into the organelle or under what conditions they are active.

One possible mechanism for matrix-localized protein phosphorylation is that the modification of select mitochondrial proteins occurs outside of the mitochondrion. Our data support a model in which phosphorylation outside of mitochondria influences Hadh import rate and downstream protease processing. In plants, phosphorylation of the targeting sequences of chloroplast-bound proteins can alter their import efficiency by promoting or disrupting molecular associations with the import complex itself, or with chaperones that facilitate import^66,67^. Consistently, phosphorylation of mitochondrial precursors such as GSTA4-4^68^, CYP2B1, and CYP2E1^69^ increases their association with cytosolic chaperones (e.g. Hsp70) to promote import, while phosphorylation of the Tom22p precursor in yeast increases its association with the import complex^60^. Alternatively, precursor phosphorylation on mitochondrial proteins including CNP2^70^ and Tom40p^61^ decrease or inhibit targeting to the organelle, consistent with our data on Hadh (Fig. 6). Beyond these examples, there are clues that mitochondrial proteins may be phosphorylated by cytosolic kinases on sequences distinct from their MTS: the outer mitochondrial membrane (OMM) protein MitoNEET is phosphorylated within its N-terminus, which may affect its membrane targeting;^71^ ferrochelatase is phosphorylated at T116 by OMM-localized PKA to activate enzymatic activity and alter heme biosynthesis^72^, and PKA has also been shown to influence the import of NDUFS4 through phosphorylation of a residue near its C-terminus^73^. Notably, PKA can be anchored to the OMM through a subset of AKAP proteins^74^, and FRET-based studies suggest that PKA activation is robust at the outer mitochondrial membrane^75^, suggesting this kinase may be properly localized to phosphorylate mitochondrial-destined proteins. Importantly, these OMM-associated kinases may spuriously modify mitochondrial precursors before import and may not have regulatory capacity. Whether regulatory or spurious in nature, these data suggest that cytosolic phosphorylation of mitochondria-bound proteins can affect their import and function through multiple mechanisms. Furthermore, the possibility that phosphorylation affects organellar targeting across species—including yeast, plants, and mammals—leads us to propose that this may be a widespread, but underappreciated, mode of regulation for organelle-bound protein precursors.

After import into the organelle, dephosphorylation is presumably required for proper processing and maturation. This model was suggested for phosphorylated chloroplast precursor proteins^67^, but a candidate phosphatase has not yet been identified. Plants have multiple *Pptc7* paralogs^76^, with a subset predicted to be chloroplast-localized, suggesting that Pptc7 paralogs likewise may mediate these functions in plants. It is also notable that Timm50 has a vestigial phosphatase domain^77^, which perhaps once functioned to dephosphorylate these proteins before matrix import. In most organisms, Timm50 does not possess critical catalytic residues within its phosphatase domain^77^; however, in select lower organisms, such as the parasitic *T. Brucei*, Timm50 (called TbTim50) retains phosphatase activity^78^. In this species, ablation of catalytic phosphatase residues alters the stability of mitochondrial proteins such as VDAC^78^, suggesting functional relevance. It is tempting to speculate that Timm50, and ultimately Pptc7, have evolved to coordinate dephosphorylation of proteins targeted to the mitochondrion.

Overall, our data add to a growing narrative that mitochondrial PTMs are widespread and impactful. It remains largely unclear whether the bulk of these PTMs are regulatory in nature, or are instead adventitious and unintentionally disruptive, and thus merely need to be removed to maintain optimal mitochondrial function. It is intriguing that our work on phosphorylation draws parallels to mitochondrial acylation, where the enzymes tasked with removing PTMs are better understood and seem to bear the larger metabolic managerial burden. In this regard, it is further noteworthy that mice lacking the mitochondrial deacetylase *Sirt3* exhibit similar phenotypes to the *Pptc7* KO mice, albeit to a lesser extent and not until they are stressed^79,80^. However, our data also suggest that some mitochondrial phosphorylation events have important regulatory potential. First, the fact that *Pptc7* KO mice develop in utero, but fail to thrive during the specific perinatal stage may be consistent with a regulatory switch of sorts. Second, our data lead us to speculate that select mitochondrial proteins are phosphorylated in the cytosol, and then dephosphorylated by phosphatases within the organelle. In this model, proteins would be available to various cytosolic kinases, whose classic signaling functions may then serve to direct proteins to mitochondria or to alter their import rates. Regardless of this regulatory potential, our data demonstrate that Pptc7 is required for surviving the perinatal transition, and show that protein dephosphorylation within the mitochondrial matrix is essential for mammalian development. Further studies will be required to understand the full repertoire of Pptc7 substrates, the potential regulation of this phosphatase, and the physiological consequences of *Pptc7* disruption in conditions beyond birth.

## Methods

### Creation of the Pptc7 knockout mouse model

A Pptc7 knockout strain was generated using CRISPR-Cas9 technology in the C57BL/6J (B6) strain of *Mus musculus* [NCBItax:10090]. Mutational details are provided at MGI; Pptc7^em1Pag^ at MGI:6094244, and Pptc7^em2Pag^ at MGI:6143811. The second and third coding exons of Pptc7 were targeted for genome editing using two target sequences to maximize specificity (all predicted off-target sites had (i) at least 3 mismatches, with at least 1 mismatch in the 12 bp seed region or (ii) 2 mismatches in the seed region). Target sequences for both exons, as well as primers associated with generation of this model, can be found in Supplementary Table 1. In vitro transcription template was generated by overlap-extension PCR with one oligo carrying a 5′ T7 adapter, the target sequence, and a portion of the common gRNA sequence, and the other oligo carrying the antisense common gRNA sequence using oligonucleotides listed in Supplementary Table 1. In vitro template was column-purified and in vitro transcribed with the MEGAshortscript kit (Thermo-Fisher), and the resultant gRNA was cleaned with the MEGAclear kit (Thermo-Fisher). For injection-grade purification, gRNA was ammonium acetate purified, washed with 70% ethanol, and resuspended in injection buffer. One-cell fertilized C57BL/6 J embryos derived from mice obtained from Jackson laboratories were microinjected with a mixture of both gRNAs (25 ng/ul each) and Cas9 protein (PNA Bio, 40 ng/ul), and then implanted into pseudopregnant B6 recipients. Tail DNA was harvested from resultant pups at weaning and used for genotyping.

### Breeding, care, and selection of mice for experiments

All animal work complied with ethical regulations for animal testing and research, and was done in accordance with IACUC approval by the College of Agricultural and Life Sciences (CALS) Animal Care and Use Committee at the University of Wisconsin–Madison (protocol/animal welfare assurance #A3368-01). The Pptc7^*-/-*^ *Mus musculus* strain was generated via CRISPR and is registered with MGI under the names C57BL/6J-Pptc7^em1Pag^ (accession number MGI:6094249) and C57BL/6J-Pptc7^em2Pag^ (accession number MGI:6143812); two strains are registered as two independently segregating Pptc7^*−/−*^ alleles were generated in our founder mouse—one with a 4 bp deletion in exon 2 (hereby called E2; MGI:6094244), and one with a 1 bp deletion in exon 3 (hereby called E3; MGI:6143811). As the phenotypes of all Pptc7-null genotypes were identical (see Supplementary Fig. 1), all genotypes are collectively annotated as “Pptc7^*−/−*^ mice” and not by their specific alleles unless noted in the text or figure legends. The C57BL/6J wild type strain (Jackson Laboratories) was used for the generation of the Pptc7-CRISPR strain as well as for outbreeding. Mice were housed on a 12-h light:dark cycle, and group housed by strain and sex under temperature- and humidity-controlled conditions and received ad libitum access to water and food. Upon weaning, mice were maintained on a standard chow diet (Formulab 5008; 17.0% kcal fat; 56.5% carbohydrate; 26.5% protein) Strains were housed within the same vivarium throughout the duration of the study. Studies were performed with P0 mouse pups, sacrificed within 24 h of their birth unless otherwise noted. Sex was not determined for experiments as males and females are indistinguishable at P0. Littermates were typically selected according to genotype and randomly assigned to experimental groups. For further information on selection of mice for experimental procedures, please see Supplementary Methods.

### Genotyping analysis

Tail tips were isolated from each mouse and used as a source of genomic DNA (gDNA). Tails were resuspended in 600 µl of Genomic Lysis Solution (20 mM Tris, pH 8.0, 150 mM NaCl, 100 mM EDTA, 1% SDS) supplemented with 3 µl concentrated proteinase K (Roche) and incubated at 55 °C overnight. After the overnight incubation, samples were cooled to room temperature for 10 min before the addition of 200 µl Protein Precipitation Solution (Qiagen). Samples were then incubated on ice for 5 min before vortexing for ~15 s. per sample. Samples were centrifuged (16,000×*g*) for 5 min to pellet precipitated proteins. The supernatant was removed and DNA was purified via isopropanol precipitation. gDNA was quantified using a Nanodrop (Thermo-Fisher) and ~300 ng of gDNA was added to each genotyping reaction. Further details regarding the genotyping strategy can be found in the Supplementary Methods, and genotyping primer sequences can be found in Supplementary Table 1.

### Next generation sequencing

The F0 founder mouse was sacrificed at 18 months of age via CO_2_ asphyxiation and tissues isolated, including brain (cerebrum), small intestine, kidney, skeletal muscle (gastrocnemius), spleen, stomach, liver, and heart, and tissues from two heterozygous F1 offspring (age 15 months, female). Genomic DNA (gDNA) was harvested from each tissue using a Qiagen DNeasy Blood & Tissue Kit (Qiagen) according to the manufacturer’s protocol. Each sample was amplified using genotyping primers for exon 2 or exon 3 as a template for NGS-compatible sequencing, and then amplified using i5/i7 compatible NGS primers, the sequences of which can be found in Supplementary Table 1. Purified amplicons were submitted to the University of Wisconsin–Madison Biotechnology Center, where libraries were prepared with guidance from Illumina’s 16s Metagenomic Sequencing Library Preparation Protocol, Part #15044223 Rev. B (Illumina), with slight modifications. Illumina dual indexes and Sequencing adapters were added. Following PCR, libraries were cleaned using a 0.9× volume of AxyPrep Mag PCR clean-up beads, and were standardized to 2 nM and pooled prior to sequencing. Paired end, 150 bp sequencing was performed using the Illumina MiSeq Sequencer and a MiSeq 300 bp (v2) sequencing cartridge. Images were analyzed using the standard Illumina Pipeline, version 1.8.2. Analysis of NGS data was performed by the University of Wisconsin (UW) biotechnology center. More details on Next Generation Sequencing and analysis can be found in the Supplementary Methods.

### Serum metabolite measurements

P0 pups were sacrificed via decapitation, and a small amount of whole blood was used to measure blood glucose using a CVS Health Advanced Blood Glucose Meter (CVS). Blood glucose levels were taken at least twice, and averaged values reported for each mouse. The remainder of the blood was collected, spun at 16.1 K×*g* for 10 min at room temperature, serum isolated and stored at −80 °C until use. Circulating insulin was assayed using the Ultra Sensitive Mouse Insulin ELISA kit (Crystal Chem) according to manufacturer’s instructions using the “Low Range Assay” protocol. Briefly, serum from *n* = 8 WT, *n* = 5 heterozygous, and *n* = 10 KO mice was thawed on ice. 5 μl of serum was assayed for each mouse and quantified relative to the standard curve generated from the kit. Lactate levels were measured in serum isolated from *n* = 9 WT, *n* = 20 heterozygous, and *n* = 7 KO mice using the Lactate Colorimetric/Fluorometric Assay Kit (BioVision) according to manufacturer’s instructions, using 5 μl of serum from each mouse quantified relative to the standard curve generated from the kit. Ketones were measured in serum isolated from newborn mice using the Ketone Bodies Kit (both R1 and R2 sets, Wako Biosciences) according to the manufacturer’s protocol. Serum from *n* = 9 WT, *n* = 13 heterozygous, and *n* = 6 KO mice was thawed on ice, and 5 μl was assayed for each mouse and quantified relative to a β-hydroxybutyrate standard curve. For all serum tests, values reported are averaged from all measurements per group, and significance was tested using a two-tailed Student’s *t*-test.

### Metabolomics and Lipidomics

Heart and liver tissues were immediately harvested and snap frozen in liquid nitrogen unless otherwise noted. Metabolites were extracted from cryo-pulverized tissue with a solvent mixture consisting of 7:2:1 HPLC grade methanol:water:chloroform; this was followed by a subsequent extraction of lipids by addition of chloroform to 50% of the final volume. Acylcarnitines were analyzed by reversed phase liquid chromatography—tandem mass spectrometry on a Q Exactive Focus operating in positive ion mode (RPLC-ESI-MS/MS). Amino acids and other polar metabolites were derivatized with methoxyamine-HCl and MSTFA and analyzed by gas chromatography—electron ionization mass spectrometry on a GC-Orbitrap (GC-EI-MS). Lipids and Co-enzyme Q (CoQ) intermediates were analyzed by RPLC-ESI-MS/MS in alternating positive and negative ion mode, with PRM targets for selected CoQ intermediates (for more details, see Supplementary Methods). Acyl-carnitines and CoQ intermediates were quantified from peak area using Thermo’s Tracefinder application. For details regarding GC and lipid feature identification, see Supplementary Methods.

### Electron microscopy

Tissues were harvested from P0 pups and were quickly washed in PBS and fixed in ~5 ml of fixation buffer (2.5% glutaraldehyde, 2.0% paraformaldehyde in 0.1 M sodium phosphate buffer, pH 7.4) overnight @ 4 °C. The tissue was then post fixed in 1% Osmium Tetroxide in the same buffer for 3 h @ RT, and the samples were dehydrated in a graded ethanol series, then further dehydrated in propylene oxide and embedded in Epon epoxy resin. Semi-thin (1 µm) sections were cut with a Leica EM UC6 Ultramicrotome and collected on 200 mesh copper grids. Sections were and contrasted with Reynolds lead citrate and 8% uranyl acetate in 50% EtOH. Ultrathin sections were observed with a Philips CM120 electron microscope and images were captured with an AMT BioSprint side-mounted digital camera using AMT Capture Engine software.

### Enzyme assays

Citrate synthase activity was assayed using genotype verified tissues (*n* = 3 for wild type and 4 for *Pptc7* knockout tissues); for details on the activity assay, please see Supplemental Methods. Error was calculated using standard deviation, and significance was calculated using a two-tailed Student’s *t*-test. For details on the Hadh activity assay, please see Supplemental Methods. Briefly, recombinant human HADH (Entrez Gene #3033) or HADH site-specifically phosphorylated at S13 (see “Phosphoserine incorporation of recombinant proteins using cell-free protein synthesis” for more details) were generated with a C-terminal FLAG tag using cell-free protein synthesis. Proteins were immunoprecipitated (IPed) using M2-FLAG antibody-conjugated magnetic beads, eluted in FLAG peptide, and HADH activity was assayed using 2.5 μl eluate from each IP (corresponding to ~200 ng recombinant protein per reaction). Error was calculated using standard deviation, and significance was calculated using a two-tailed Student’s *t*-test.

### Quantitative multiplexed proteomics and phosphoproteomics

Protein from lysed, homogenized mouse tissues was digested into tryptic peptides (for specific details, please see Supplemental Methods). This yielded sufficient material to label 0.35 mg of each heart sample and 0.5 mg of each liver sample with tandem mass tags (TMT10plex Isobaric Label Reagent Set, Thermo-Fisher). Labeling was performed following manufacturer recommended protocols except for the peptide:label ratio, which was changed to 0.5:0.8::mg:mg. Equal amounts of sample were combined for each 10-plex experiment following the validation of labeling efficiency (>95% labeling of N-terminal amines). Phosphopeptide enrichment with titanium chelation (Ti-IMAC, ReSyn Biosciences) was performed (see Supplemental Methods for further information). The depleted sample was saved for protein quantitation. 500 µg of each depleted sample and the entirety of each phosphopeptide enrichment were separated over an Acquity BEH C18 reverse phase column (130 Å pore size, 1.7 µm particle size, 2.1 × 100 mm, Waters Corp) held at 60 °C using a Dionex Ultimate 3000 uHPLC (600 µL/min flow rate, Thermo-Fisher) running with basic mobile phases. Resulting fractions were analyzed on a q-LTQ-OT hybrid mass spectrometer (Orbitrap Fusion Lumos, Thermo-Fisher) operated under data-dependent acquisition following nano-LC separation and electrospray ionization. MS1 survey scans were performed in the Orbitrap (60 K resolution, AGC – 1e6, 50 ms max injection time). Product ion scans following HCD fragmentation (35% NCE) were also performed in the Orbitrap (60 K resolution, AGC—2e5, 118 ms max injection time). Monoisotopic precursor selection and dynamic exclusion (60 s) were enabled. Thermo RAW files were searched with the Open Mass Spectrometry Search Algorithm (OMSSA) within the Coon OMSSA Proteomic Analysis Software Suite (COMPASS) (for database details, see Supplemental Methods). TMT labeling was imposed as a fixed modification at lysines and N-termini and variable at tyrosines. Additionally, phosphorylation of serine, threonine, and tyrosine, as well as accompanying neutral losses, were set as variable modifications for enriched samples. Search results were filtered to a false discovery rate of 1% at the peptide and protein levels. Sites of phosphorylation were considered localized if given a localization score > 0.75 by the PhosphoRS module within COMPASS. Phosphopeptide intensities were normalized to the total reporter ion intensity at the protein level, as well as, protein mean-normalized fold change. An associated *p*-value was calculated using Student’s *t*-test assuming equal variance. Multiple hypothesis testing was performed by Benjamini–Hochberg correction. The mass spectrometry proteomics data have been deposited to the ProteomeXchange Consortium via the PRIDE partner repository with the dataset identifier PXD012743. Further details regarding proteomic and phosphoproteomic sample preparation, mass spectrometry, and analysis are available in the Supplemental Methods.

### RNA isolation and qPCR

RNA was isolated using an RNeasy kit (Qiagen) per the manufacturer’s instructions. cDNA was prepared using 500 ng of total RNA using the SuperScript III First-Strand Synthesis System (Thermo-Fisher). cDNA was made exclusively with the oligo(dT) primer, and the kit was used according to the manufacturer’s protocol. cDNA was normalized, and 100 ng was used as a template for each qPCR reaction. Reactions were set up using 1× PowerSYBR master mix (Thermo-Fisher), 200 nM forward and reverse primers (each) and water to a final volume of 20 μl total. Primer sequences used in this study can be found in Supplemental Table 1. qPCR was run on a QuantStudio 6 Flex (Applied Biosystems) and data were captured using QuantStudio Real-Time PCR system software. Data were analyzed using the ΔΔCt method (*n* = 4 samples for wild type and 3 samples for knockout tissue), with error bars representing standard deviation and significance calculated using a two-tailed Student’s *t*-test.

### Cloning and site-directed mutagenesis

*Escherichia coli* strain DH5α (NEB) was used for all cloning applications and grown at 37 °C in LB media with antibiotics. *Escherichia coli* strain BL21-CodonPlus (DE3)-RIPL (Agilent) was used for all protein expression and purification purposes. For details on site-directed mutagenesis, please see Supplemental Methods.

### Mitochondrial import assays

Various strains derived from the haploid W303 (*his3 leu2 lys2 met15 trp1 ura3 ade2*) and YPH499 (*ura3-52 lys2-801_amber ade2-101_ochre trp1-Δ63 his3-Δ200 leu2-Δ1*) yeast strains were used for mitochondrial isolation for import assays. For details on the generation of the Δ*ptc7* strain was and yeast strains expressing Tim50 S104E and S104A mutations, please see Supplemental Methods. For mitochondrial isolation from yeast, a single colony of W303 yeast was incubated in 3–4 ml of YPD media overnight. 1 × 10^8^ cells were diluted into 500 ml of YEP supplemented with 3% glycerol 0.1% dextrose. Yeast were grown at 30 °C in an orbital shaker (230 rpm) for 19–20 h to a final OD of ~5–6 (with an OD of 1 = 1e7 cells). For details on mitochondrial isolation from these strains, please see Supplemental Methods. Isolated mitochondria were resuspended using wide-bore tips to a final concentration of 10 mg/ml in SEM buffer (250 mM sucrose, 1 mM EDTA, and 10 mM MOPS, pH 7.2), snap frozen in liquid nitrogen, and stored at −80 °C until use in import assays. Recombinant Mdh1p, Atp2p, and cytochrome b_2_-(167)_∆19_-DHFR were generated using the quick TnT® Quick Coupled Transcription/Translation System (Promega) supplemented with ^35^S-Methionine and Cysteine (EasyTag EXPRESS35S protein labeling mix, Perkin Elmer) according to the manufacturer’s instructions. Reactions were terminated by placing reactions on ice until use in mitochondrial import assays. For mammalian targets, radiolabeled HADH (WT, S13A, S13E) precursor proteins were generated by in vitro transcription/translation reactions in rabbit reticulocyte lysate in the presence of ^35^S-Methionine. Mitochondria were isolated from HEK293T cells (for additional details, see Supplemental Methods), and for import assays, mitochondria were resuspended in import buffer (250 mM sucrose, 5 mM magnesium acetate, 80 mM potassium acetate, 10 mM sodium succinate, 20 mM HEPES-KOH, pH 7.4) supplemented with 1 mM DTT and 5 mM ATP. Where indicated, the membrane potential was dissipated prior to the import reaction by addition of 8 μM antimycin A, 1 μM valinomycin and 20 μM oligomycin (AVO). Import reactions were started by addition of radiolabeled precursor proteins followed by incubation at 37 °C for indicated times. Reactions were stopped by addition of AVO and non-imported precursor proteins removed by digestion with Proteinase K. Mitochondria were re-isolated by centrifugation at 10,000 × *g* for 10 min at 4 °C and washed once in import buffer. Samples were analyzed on SDS-PAGE followed by digital autoradiography. Uncropped and unprocessed autoradiographs from the main text can be found in Supplementary Fig. 7 in the Supplementary Information. Images were quantified using ImageJ.

### MPP processing assay

MPP processing assays using soluble mitochondrial extracts from mouse liver were performed^55^. In short, mitochondria isolated from mouse liver were lysed in digitonin and the obtained extract incubated with radiolabeled precursor proteins. Samples were incubated for various time points at 37 °C and reactions stopped by addition of 4× Laemmli buffer containing 2% (v/v) β-mercaptoethanol. Samples were analyzed by SDS-PAGE and blotted onto PVDF membranes followed by autoradiography and immunodetection. For information on antibodies used, refer to “Western blotting and SDS-PAGE”.

### Generation of phosphoserine protein synthesis reagents

Cell-free protein synthesis (CFPS) was used to generate phosphoserine incorporation. The bacterial strain C321.ΔA.Δserb.Amp^S^ was a gift from Jesse Rinehart (Addgene #68306) and was used to generate lysates for the CFPS reactions^50^. The bacteria were transformed with SepOTSλ, a gift from Jesse Rinehart (Addgene plasmid #68292) to allow for generation of phosphoserine-incorporated recombinant proteins. For specific details regarding the generation of bacterial lysates using this system, please see Supplementary Methods.

### Phosphoserine incorporation of recombinant proteins

CFPS reactions were performed as described by Oza et al.^50^:  Reactions contained 30% v/v cell extract supplemented with 1.2 mM ATP*, 0.85 mM each of GTP*, UTP*, and CTP* (pH 7–7.2); 34 μg/mL folic acid*; 170 μg/mL of *E. coli* tRNA mixture*; 13.3 μg/mL plasmid (maxiprepped); 100 μg/mL T7 RNA polymerase; 2 mM each of standard amino acids^†^ (omitting methionine and cysteine in S35 experiments); 0.33 mM NAD*; 0.27 mM coenzyme A*; 1.5 mM spermidine*; 1 mM putrescine*; 4 mM oxalic acid*; 130 mM potassium glutamate^‡^; 10 mM ammonium glutamate^‡^; 12 mM magnesium glutamate^‡^; 33 mM phosphoenolpyruvate (pH 7), 2 mM phosphoserine, 57 mM HEPES pH 7*. Some reagents can be pre-mixed, aliquoted, and stored at −20 °C (*pre-mix, ^†^amino acid mix; for details on preparation, see Supplementary Methods; ^‡^salt mix at pH 7 with KOH) and the final reaction should be at pH 6.5–7. CFPS reactions were initiated by thoroughly mixing the cell extract, incubated for 17–20 h at 30 °C, and performed in 15, 30, or 50 uL reactions.

### PPTC7 and Ptc7p protein purification and phosphatase assays

N41-Ptc7p and N32-PPTC7 (both N-terminal truncations of 41 and 32 amino acids, respectively, of *S. cerevisiae* Ptc7p and *H. sapiens* PPTC7) and catalytically inactive mutants were prepared by expressing His_8_-MBP-tev-Ptc7p^NΔ41^ and His_8_-MBP-tev-PPTC7^NΔ32^ and associated mutants in *E. coli* (BL21[DE3]-RIPL strain) by autoinduction^17^. Cells were isolated and resuspended in lysis buffer (100 mM HEPES pH 7.2, 300 mM NaCl, 5 mM BME, 0.25 mM PMSF, 1 mg/mL lysozyme (Sigma), and 7.5% glycerol) Cells were lysed by sonication (4 °C), clarified by centrifugation (15,000×*g*, 30 min, 4 °C), and mixed with cobalt IMAC resin (Talon resin) for one hour (4 °C). Resin was washed with Wash Buffer (20 bed volumes: Lysis buffer without lysozyme) and His-tagged protein was eluted with Elution Buffer (50 mM HEPES pH 7.2, 150 mM NaCl, 5 mM BME, 5% glycerol 100 mM imidazole). Eluted protein was concentrated with a 50-kDa MW-cutoff spin filter (Merck Millipore Ltd.) and exchanged into Storage Buffer (50 mM HEPES pH 7.2, 150 mM NaCl, 5 mM BME, 5% glycerol). His_8_-MBP- PPTC7 ^NΔ32^ or His_8_-MBP-Ptc7p^N41^ was incubated with TEV protease (1:50, TEV/fusion protein, mass:mass) for 1 h (25 °C), then incubated with cobalt IMAC resin for 1 h (4 °C). Cleaved protein was collected, concentrated with a 10-kDa MW-cutoff spin filter, and exchanged into Storage Buffer. Protein was aliquoted, frozen in N_2_, and stored at −80 °C. Phosphatase assays were run in 10 μl total volume comprised of 50 mM Tris, pH 8.0, 5 mM MnCl_2_, 5 μl of site-specifically phosphorylated recombinant protein (~400 ng substrate) and ~200 ng active (wild type) or catalytically inactive (D/A mutants) phosphatase per reaction. Reactions were allowed to proceed for 30 min at room temperature, after which they were terminated through the addition of sample buffer to 1×, boiled at 95 °C, and run on PhosTag gels to determine dephosphorylation efficiency.

### Transfection and protein expression in 293 cells

293 cells were cultured in DMEM (high glucose, no pyruvate, Thermo-Fisher) supplemented with 10% heat inactivated fetal bovine serum (FBS) and 1× penicillin/streptomycin. Cells were grown in a temperature-controlled CO_2_ incubator at 37 °C and 5% CO_2_. Cells were subcultured using 0.05% trypsin-EDTA every 2–3 days. For transfections, cells were split to ~40% confluence on Day 1. On Day 2, cells were transfected with 7.5 µg Maxiprep-purified plasmid supplemented with 20 µg linear polyethylenimine (PEI, PolySciences). After 48 h, cells were collected or fixed for downstream applications.

### Fluorescence microscopy

Glass cover slips (#1.5) were sterilized and placed into single wells of 12 well plates. 293 cells were seeded at ~60% confluence and transfected with plasmids encoding GFP or a construct expressing an N-terminal HADH-GFP fusion (expressing the first 50 amino acids of HADH fused to GFP). 20 h after transfection, cells were labeled with 25 nM MitoTracker Red CMXRos for 15 min at 37 °C. Cells were then washed 3x with PBS, and fixed using 4% paraformaldehyde in PBS for 15 min at room temperature. After fixation, cells were washed 3× with PBS, and nuclei were stained for 5 min with 2 μg/ml [final c] of Hoechst. After nuclear staining, cells were washed again 3× with PBS and mounted to glass slides using ProLong Diamond reagent overnight in the dark, per manufacturer’s instructions. Cells were imaged on a Nikon A1R-SI + confocal microscope using a ×60 oil-based objective using constant settings (e.g. laser intensity) across all images acquired. Images were collected in all three fluorescent channels using NIS element software.

### Western blotting and SDS-PAGE

Yeast total cell lysates were generated using Yeast Lysis Buffer (YLB; 100 mM Tris (pH = 7.4), 1% (v/v) Triton X-100, 1 mM EDTA, 1 mM PMSF). To generate lysate from mouse heart and liver, snap frozen tissues were thawed on ice, resuspended in Lysis Buffer A (LBA; 20 mM Tris, pH 8.0, 137 mM NaCl, 1% NP-40), and homogenized using a motorized Potter Elvehjem homogenizer at 1500 rpm. To generate lysate from cells, cells were washed 3× with PBS, LBA was added to cultured plates on ice and cells collected. After generating all lysates, samples were clarified by centrifugation at 16,000×*g* for 10 min at 4 °C, snap frozen in liquid nitrogen, and stored at −80 °C until use. All lysates were quantified via BCA, normalized, and 20–40 μg run for each blot. Antibodies used in this study include anti-FLAG-M2 (Sigma #F1804; dilution 1:5000), anti-VDAC (Abcam #ab18988, dilution 1:2000), anti-OxPhos (Abcam #ab110412, dilution 1:2000), anti-GRP75 (Abcam #ab2799, dilution (1:500), anti-PMPCB (dilution 1:250), anti-Tom70p (dilution 1:500) and anti-Tom20p (dilution 1:2000) (all developed and validated by Vogtle and/or Meisinger lab), anti-Por1p (Abcam #ab110326, dilution 1:1000), and anti-Tubulin (Abcam #ab59680; dilution 1:2000). Uncropped and unprocessed western blots from the main text can be found as Supplementary Fig. 7 in the Supplementary Information, and all uncropped and unprocessed blots are available as Source Data.

### Quantification and statistical analysis

See each individual method for the associated statistical analysis. Unless otherwise noted in the associated figure legend, all measurements reported were taken from distinct samples.

### Reporting summary

Further information on research design is available in the Nature Research Reporting Summary linked to this article.

## Supplementary information

Supplementary Information

Peer Review File

Supplementary Dataset 1

Supplementary Dataset 2

Reporting Summary

## Data Availability

The source data underlying Figs. 2f–h, 5b, d, g, 6c–e, g–h and Supplementary Figs. 2f–I, 3d, h, 5a, f, g, j, m, o, and 6a–d, g–I, k are provided as a Source Data file. The source data for proteomic and phosphoproteomic analyses are provided as Supplementary Datasets 1 and 2. The mass spectrometry proteomics data have been deposited to the ProteomeXchange Consortium via the PRIDE partner repository with the dataset identifier PXD012743. Other datasets generated during and/or analyzed during the current study are available from the corresponding author on reasonable request.

## Source data

Source Data
