# Supplementary material for: Pptc7 is an essential phosphatase for promoting mammalian mitochondrial metabolism and biogenesis
Source: Nat Commun. 2019 Jul 19;10:3197. doi: 10.1038/s41467-019-11047-6 (PMC6642090; doi:10.1038/s41467-019-11047-6)
Supplement: Supplementary file 1 — Supplementary Information [file 41467_2019_11047_MOESM1_ESM.pdf]

SUPPLEMENTARY INFORMATION

Pptc7 is an essential phosphatase for promoting mammalian mitochondrial metabolism and biogenesis

Niemi et al.

SUPPLEMENTARY FIGURES

Supplementary Figure 1

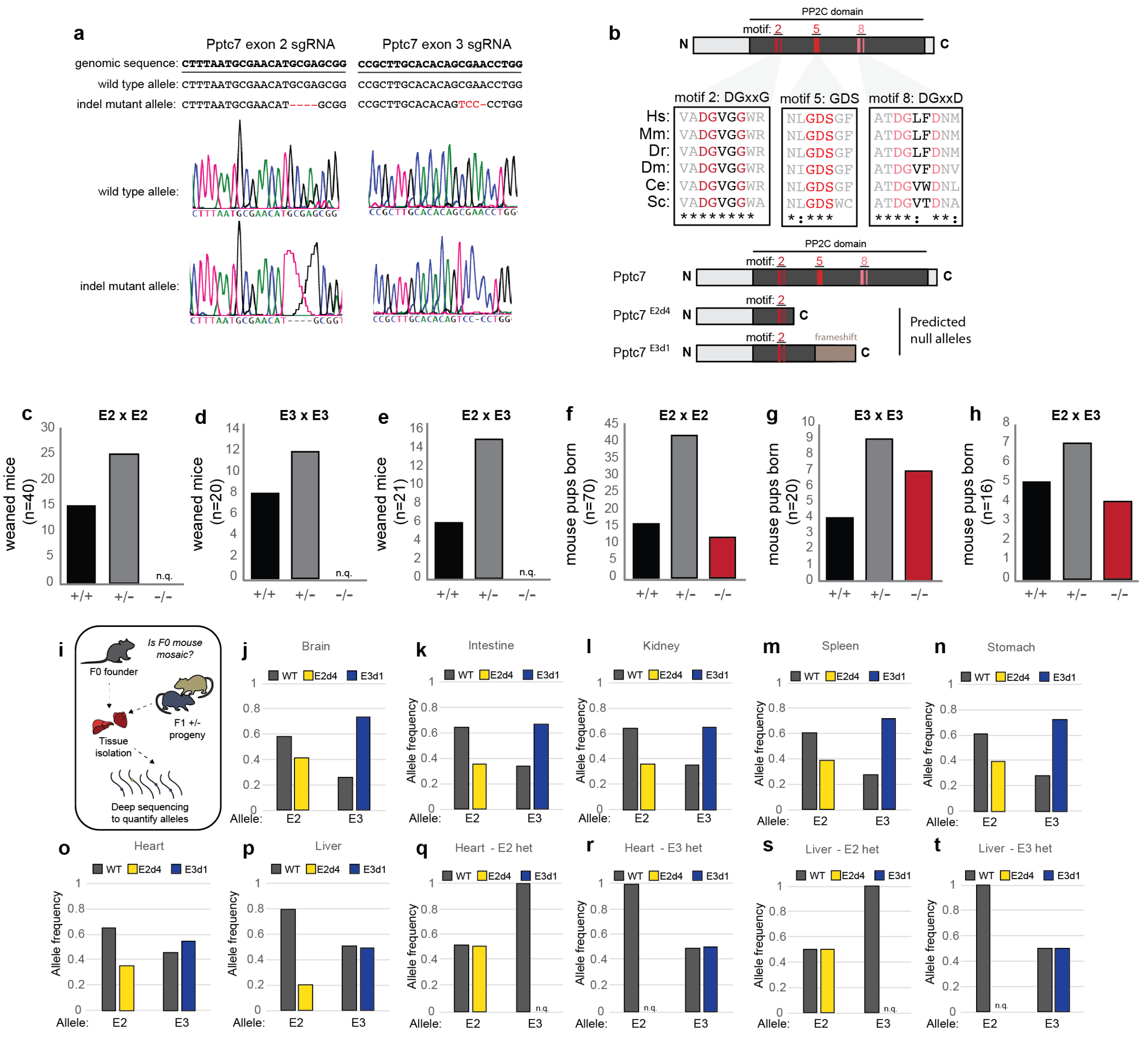

Supplementary Figure 1: Global, CRISPR-mediated knockout of Pptc7 causes perinatal lethality. a. Sequencing results show the wild type (WT) (top) and mutant (bottom) alleles under the sgRNA at exons 2 and 3 of Pptc7. b. Schematic of Pptc7 domains and key catalytic motifs (red) in WT protein (top), the E2 mutant allele-encoded protein (middle) and the E3-mutant encoded allele (bottom). Both indels cause premature stop codons and/or significant frameshifts that disrupt key PP2C phosphatase motifs. 1c.-e. Interbreeding *Pptc7* heterozygous mice carrying the E2 allele (n=40) (c.), the E3 allele (n=20) (d.), or interbreeding E2 X E3 null alleles (n=21) (e.) did not produce knockout mice at weaning (~2 weeks of age). 1f.-h. Interbreeding *Pptc7* heterozygous mice carrying the E2 allele (n=70) (f.), the E3 allele (n=20) (g.), or interbreeding the null alleles (n=16) (E2 x E3) (h.) produced knockout mice at Mendelian ratios at birth (pups

genotyped at P0). i. To determine if the founder mouse was a mosaic, we performed next generation sequencing on tissues to determine allele frequencies for WT and E2 or E3 mutant alleles. j.-p. Allele frequencies seen in the founder mouse in brain (j.), intestine (k.), kidney (l.), spleen (m.), stomach (n.), heart (o.), and liver (p.). WT alleles are shown in dark gray; E2 mutant alleles shown in yellow; E3 mutant alleles shown in blue; all alleles quantified in frequency (e.g. 100% = 1 and fractions thereof). q.-t. Allele frequencies of heterozygous F1 progeny in heart (q., r.) and liver (s., t.) showing expected allele distributions for E2 mutant mice (q., s.) and E3 mutant mice (r., t.).

Supplementary Figure 2

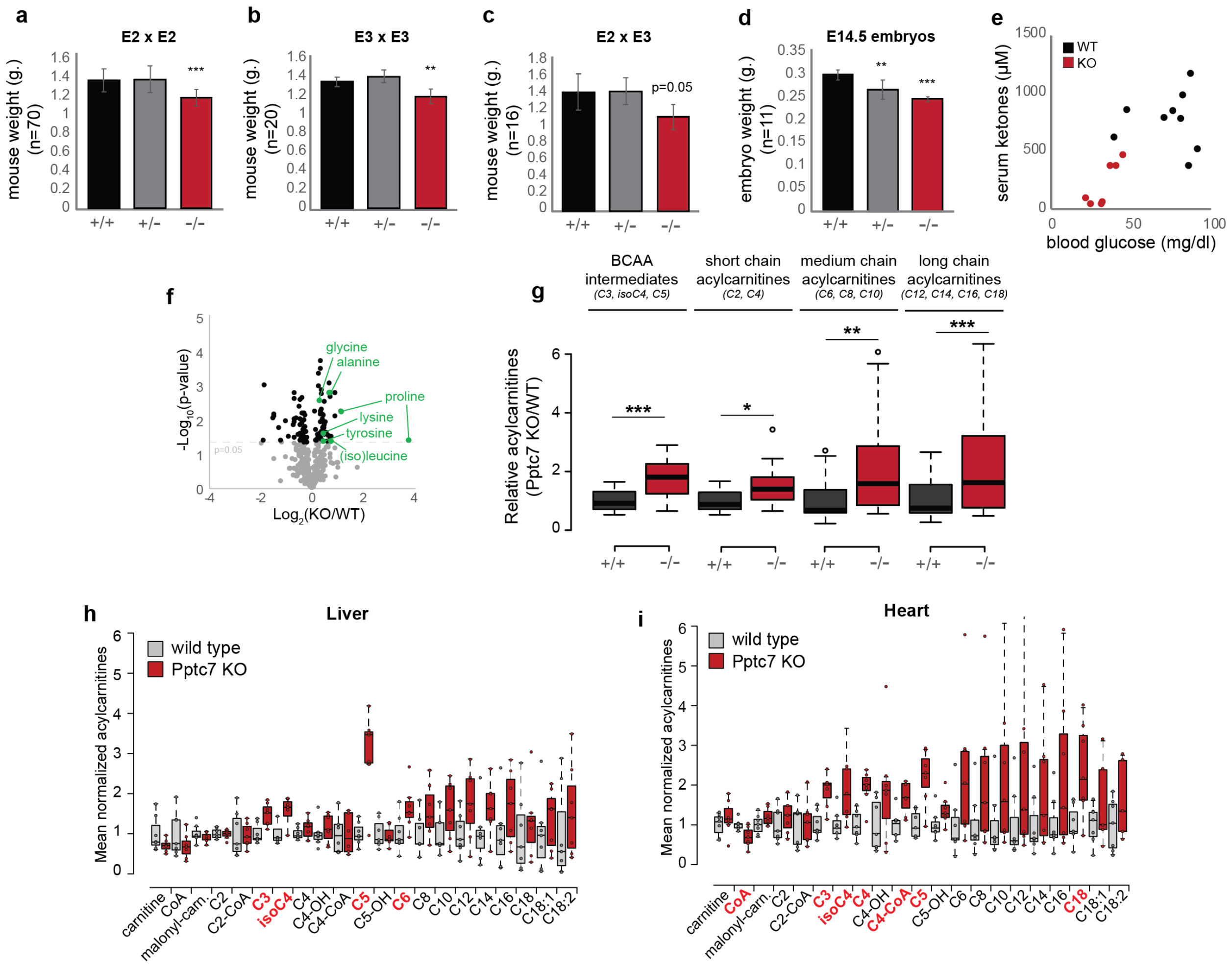

Supplementary Figure 2: *Pptc7*-null mice have defects associated with inborn errors of metabolism. a.-c. Interbreeding *Pptc7* heterozygous mice carrying the E2 allele (n=70) (a.), the E3 allele (n=20) (b.), or interbreeding E2 X E3 null alleles (n=16) (c.) shows that knockout of *Pptc7* causes decreased weight in pups of all genotypes. d. Embryos (n=11 total; n=4 WT, n=4 het, n=3 knockout (KO) pups per genotype) isolated at E14.5 show that knockout of *Pptc7* decreases weight, showing intrauterine growth restriction. e. Matched analysis of blood glucose levels (x-axis) and serum ketone levels (y-axis) reveals that knockout pups (red) have hypoketotic hypoglycemia (unlike WT pups, shown in black). f. Metabolite analysis of *Pptc7* KO heart tissue (n=7) shows elevations in select amino acids relative to WT heart tissue (n=7), as labeled. g. Acylcarnitine analysis of *Pptc7* WT and KO heart tissue (n=7 tissues per genotype for each acylcarnitine measured) shows similar defects as those found in liver; elevated BCAA intermediates as well as broad increases in FAO intermediates. h., i. Individual acylcarnitines identified in liver (h.) and heart (i.); (n=7 tissues per genotype for each acylcarnitine measured

except in heart tissue, where n=5 for the KO samples for C18:1 and 18:2). Significantly changing species ($p<0.05$, two-tailed Student's t-test) are labeled in bold red on the x-axis. For bar graphs, error bars represent standard deviation. For box plots, center lines show the medians; box limits indicate the 25th and 75th percentiles as determined by R software; whiskers extend 1.5 times the interquartile range from the 25th and 75th percentiles, and outliers are represented by dots. Significance for all panels calculated by a two-tailed Student's t-test; * = $p<0.05$, ** = $p<0.01$, *** = $p<0.001$, n.s. = not significant; $p>0.05$. Source data for panels f.-i. are provided as a Source Data file.

Supplementary Figure 3

a **Heart - non-mitochondrial protein GO analysis**

GO biological process complete	GO Term ID	Fold enrich	p-value
glutamine metabolic process	(GO:0006541)	23.66	4.79E-02
tRNA aminoacylation - translation	(GO:0006418)	17.54	5.05E-04
tRNA aminoacylation	(GO:0043039)	16.35	8.20E-04
amino acid activation	(GO:0043038)	15.98	9.56E-04
protein import into nucleus	(GO:0006606)	12.52	3.82E-05
import into nucleus	(GO:0051170)	11.91	6.10E-05
nucleosome assembly	(GO:0006334)	9.72	2.92E-02
chromatin assembly	(GO:0031497)	9.67	1.80E-03
mRNA transport	(GO:0051028)	9.6	4.69E-04
DNA packaging	(GO:0006323)	9.25	1.08E-05
DNA conformation change	(GO:0071103)	8.63	4.77E-07
RNA localization	(GO:0006403)	8.48	8.34E-06
RNA transport	(GO:0050658)	8.41	1.23E-04
nucleic acid transport	(GO:0050657)	8.41	1.23E-04
establishment of RNA localization	(GO:0051236)	8.29	1.44E-04

b **Liver - non-mitochondrial protein GO analysis**

GO biological process complete	GO Term ID	Fold enrich	p-value
regulation of RNA splicing	(GO:0043484)	6.29	9.25E-03
regulation of mRNA processing	(GO:0050684)	6.11	1.22E-02
nucleocytoplasmic transport	(GO:0006913)	5.54	4.89E-03
nuclear transport	(GO:0051169)	5.54	4.89E-03
ribonucleoprotein complex assembly	(GO:0022618)	5	1.54E-02
ribonucleoprotein complex organization	(GO:0071826)	4.7	3.04E-02
regulate intracellular protein transport	(GO:0033157)	4.57	4.09E-02
mRNA processing	(GO:0006397)	4.46	2.55E-05
RNA splicing	(GO:0008380)	4.27	4.61E-03
ribonucleoprotein complex biogenesis	(GO:0022613)	4.18	1.74E-04
mRNA metabolic process	(GO:0016071)	4.01	1.14E-05
covalent chromatin modification	(GO:0016569)	3.83	3.81E-02
intracellular protein transport	(GO:0006886)	3.4	5.75E-06
chromatin organization	(GO:0006325)	3.23	1.64E-03
RNA processing	(GO:0006396)	3.1	4.60E-04

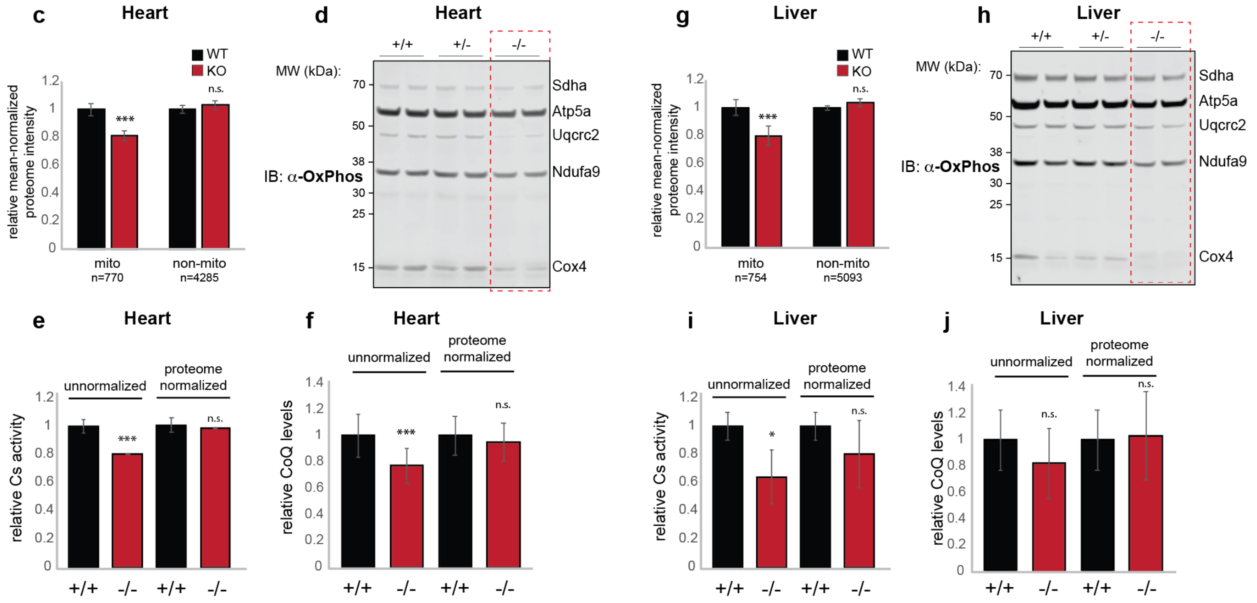

Supplementary Figure 3: Loss of *Pptc7* selectively decreases mitochondrial content. a., b. GO term analysis for non-mitochondrial proteins significantly altered in *Pptc7* KO heart (a.) and liver (b.) relative to WT tissue. c. Relative mean normalized mitochondrial proteome (n=770 quantified proteins) is significantly downregulated in heart, whereas the non-mitochondrial proteome (n= 4285 quantified proteins) does not show a significant difference between WT and KO tissues. d. Western blots of n=2 lysates generated from WT (+/+), heterozygous (+/-) or knockout (-/-) heart tissue and probed for various proteins involved in oxidative phosphorylation (labeled at left) show select decreases only in knockout tissues (highlighted by dashed red box). e. Citrate synthase (Cs) activity is significantly decreased in *Pptc7* KO heart tissue relative to WT tissue (n=4 for each genotype), but when normalized to the mitochondrial proteome (quantified in c.), the values are no longer significant. f. CoQ levels are decreased in heart tissue (n=7 for each genotype), but when normalized to total mitochondrial content (quantified in c.), these values are no longer significant. g. Relative mean normalized mitochondrial proteome (n=754 quantified proteins) is significantly downregulated in liver tissue, whereas the non-mitochondrial proteome (n=5093 quantified proteins) does not show a significant difference between WT and KO tissues. h. Western blots of n=2 lysates generated from WT (+/+),

heterozygous (+/-) or knockout (-/-) liver tissue and probed for various proteins involved in oxidative phosphorylation show select decreases only in knockout liver. i. Citrate synthase (Cs) activity was decreased in *Pptc7* KO liver tissue relative to WT liver (n=4 for each genotype), but when normalized to the mitochondrial proteome (quantified in g.), the values are no longer significant. j. CoQ levels are decreased in liver tissue (n=7 for each genotype), but when normalized to total mitochondrial content (quantified in g.), these values are no longer significant. For bar graphs, error bars = standard deviation. Statistical analysis for all panels was performed using a two tailed Student's t-test; * = $p<0.05$, ** = $p<0.01$, *** = $p < 0.001$, n.s. = not significant; $p>0.05$. Source data for panels d. and h. are provided as a Source Data file.

Supplementary Figure 4

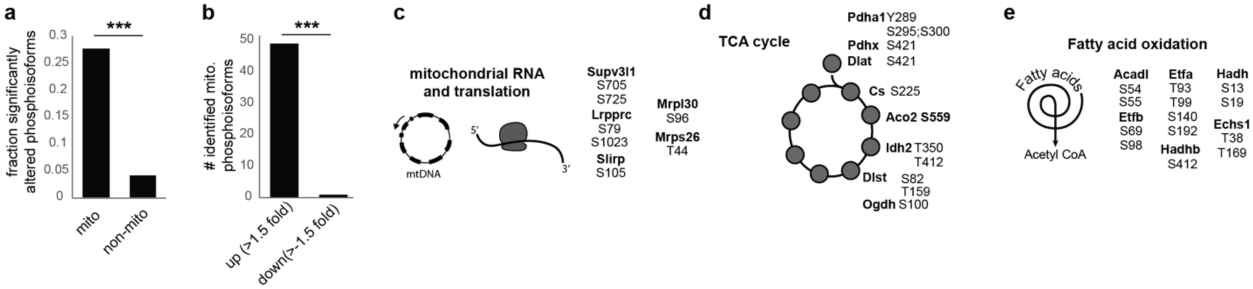

Supplementary Figure 4: Phosphoproteomic analysis reveals candidate Pptc7 substrates. a. Of changing (defined as those with statistical significance of $p<0.05$, two tailed Student's t-test) phosphopeptides identified in both heart and liver tissue, significantly more were derived from mitochondrial-localized proteins versus non-mitochondrial proteins (χ^2 test, *** = $p<0.001$). b. Of identified, changing (defined as those with statistical significance of $p<0.05$ as calculated by a two tailed Student's t-test) mitochondrial isoforms, significantly more phosphopeptides were increased (>1.5 fold) than decreased (>-1.5 fold) (χ^2 test, *** = $p<0.001$). c.-e. Phosphopeptides elevated in *Pptc7* KO tissues relative to WT tissues span multiple pathways, including mitochondrial RNA and translation (c.), the TCA cycle (d.) and fatty acid oxidation (e.). Phosphosites are listed below proteins, which are listed in bold.

Supplementary Figure 5

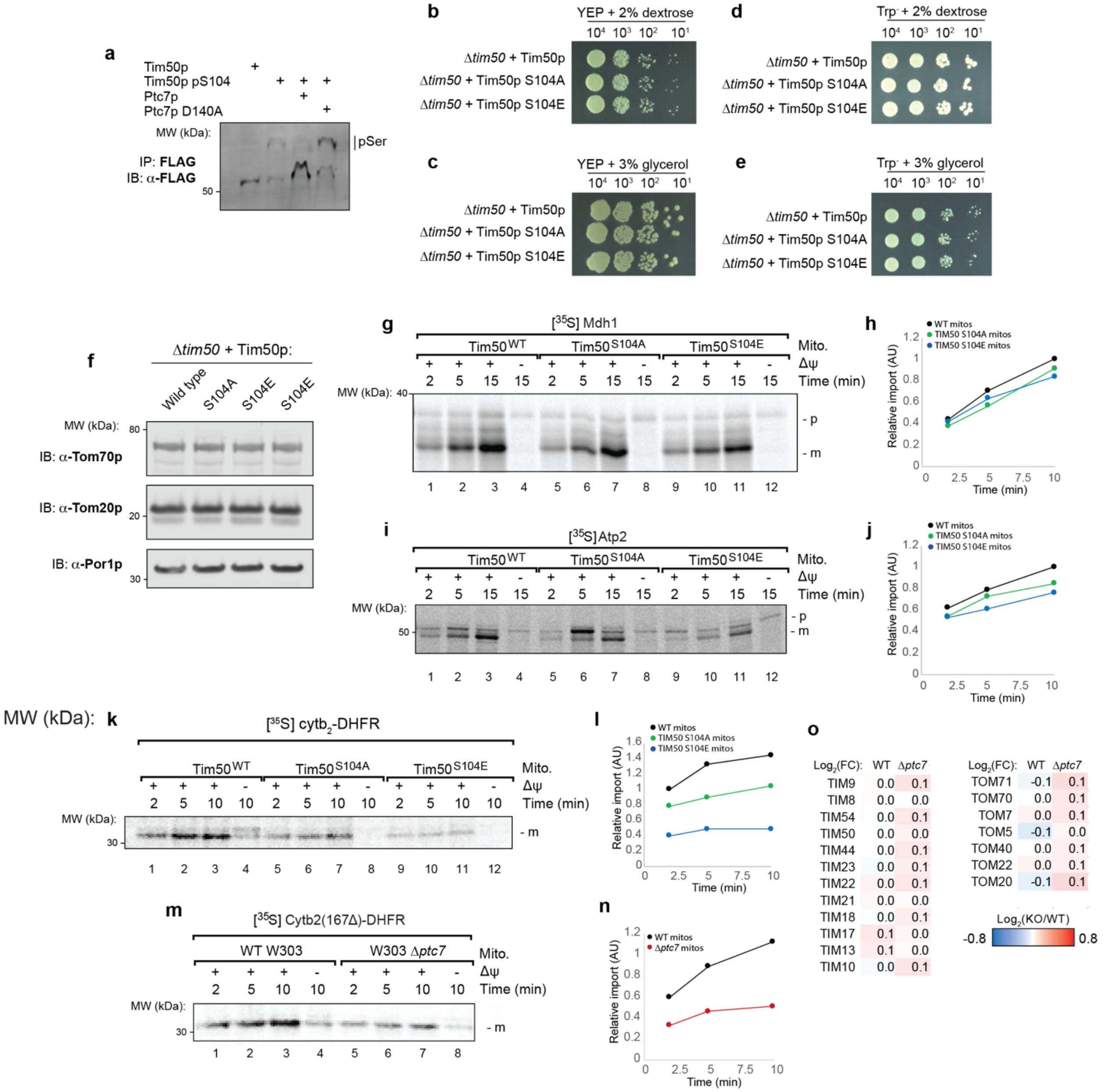

Supplementary Figure 5: Phosphorylation Timm50 decreases mitochondrial protein import a. Wild type and site-specifically phosphorylated (pS104) recombinant Tim50p were generated and phosphorylation state was validated by PhosTag gels (see pSer band shift). Addition of active Ptc7p, but not catalytically inactive D140A, promotes the dephosphorylation of Tim50p. b.-e. Δ*tim50* yeast rescued with WT, S104A, or S104E TIM50 do not show growth defects on rich media with 2% dextrose (b.), 3% glycerol (c.), or Trp drop out media containing 2% dextrose (d.), or 3% glycerol (e.). f. Western blotting for endogenous Tom70p, Tom20p, and Por1p shows no differences in TOM complex components in Δ*tim50* yeast rescued with WT,

S104A, or S104E TIM50. g.-j. Import assays in Δ*tim50* yeast mitochondria overexpressing wild type (WT) TIM50 or S104 mutants using radiolabeled Mdh1 (g.,h.) or Atp2 (i.,j.). Imported proteins result in accumulation of the mature (m) versus precursor (p) bands. Quantification of import rates is shown over time (h., j.), k. Import assays in yeast expressing endogenous TIM50 under the *GAL7* promoter. Mitochondria overexpressing wild type (WT) TIM50 or S104 mutants using the generic import cargo cytochrome b_2-(167)$_{\Delta 19}$-DHFR. l. Quantification of import rates shown in (k.). m. Import assays in Δ*ptc7* yeast mitochondria using the matrix-targeted model substrate cytochrome b_2-(167)$_{\Delta 19}$-DHFR. n. Quantification of import rates as shown in (m.). o. Heatmap of all quantified TIM and TOM complex members in WT and *ptc7*-null yeast, with scale for fold change at bottom right. Source data for panels a., f., g., i., k., and m. are provided as a Source Data file.

Supplementary Figure 6

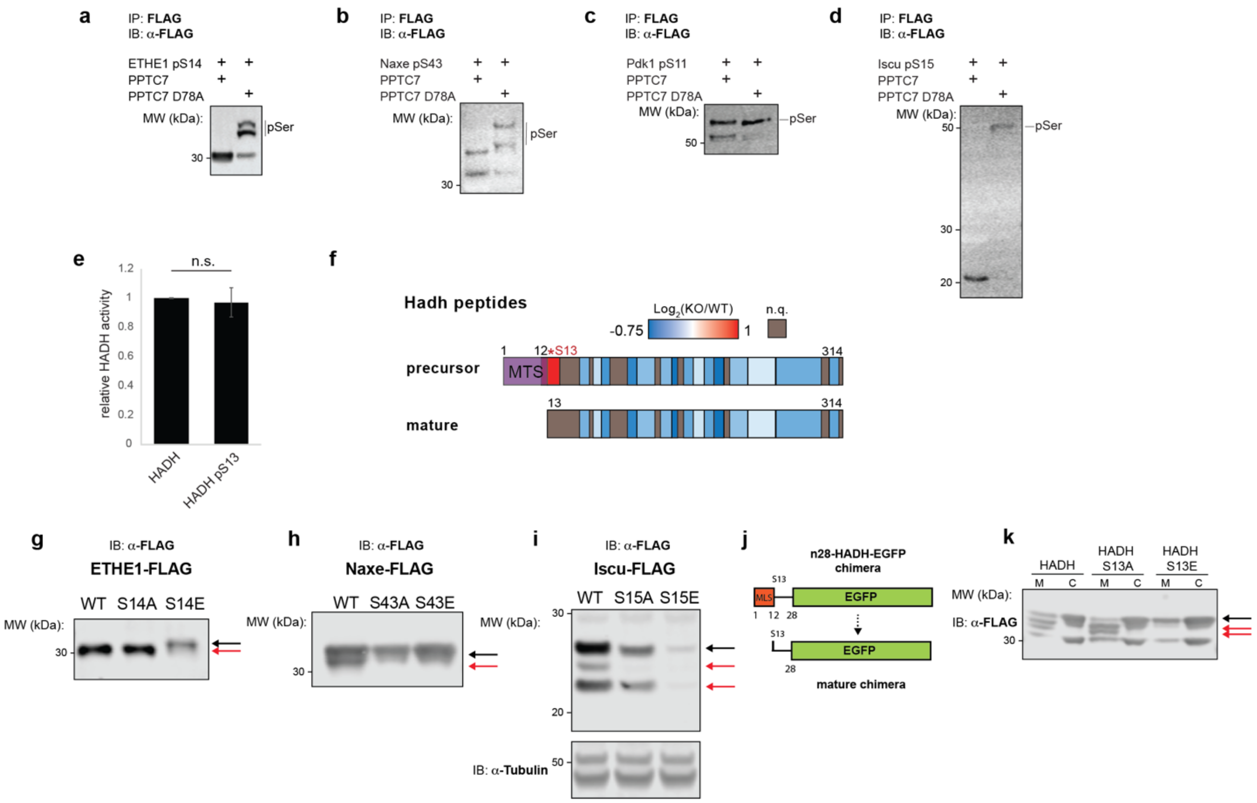

Supplementary Figure 6: Pptc7 influences import and processing by dephosphorylating MTS residues a.-d. Recombinant site-specific incorporated Ethe1 pS14 (a.), NAXE pS43 (b.), Pdk1 pS11 (c.), and Iscu pS15 (d.) can be dephosphorylated by recombinant PPTC7, but not a catalytically inactive mutant (D78A). e. HADH and site-specifically phosphorylated HADH (pS13) have similar enzymatic activity. Error bars represent standard deviation of n=3 technical replicates; statistical analysis performed using a two tailed Student's t-test; n.s. = not significant, p>0.05. f. Representation of quantified peptides on precursor (top) or mature (bottom) Hadh. Color scheme represents the relative fold change in KO/WT tissues; the only peptide to be upregulated contains MTS residues (shown overlapping with purple MTS on schematic), indicating it is from precursor but not mature protein. g.-i. Overexpression of WT, non-phosphorylatable (S13A) and phosphomimetic (S13E) phosphoMTS proteins in 293 cells. In ETHE1, S14E causes a shift in ETHE1 processing relative to S13A and WT (g.); in Naxe, S43A or S43E causes a shift in processing (h.), and in Iscu, S15E causes destabilization of the protein (i.); Tubulin blot shown as loading control (bottom panel). j. A fusion protein of the first 28 amino acids of HADH and GFP was made, expressing WT or S13 mutants. k. After overexpression of the n28-HADH-GFP construct in 293 cells, a crude mitochondrial fractionation was performed, demonstrating that processed bands (denoted by red arrows) only

occur in the mitochondrial (M) fractions, and not the cytoplasmic (C) fractions. Notably, these bands are absent in the HADH S13E lanes, which shows full length HADH S13E accumulating in the mitochondrial fraction. Source data for panels a.-d., g.-i., and k. are provided as a Source Data file.

Supplementary Figure 7

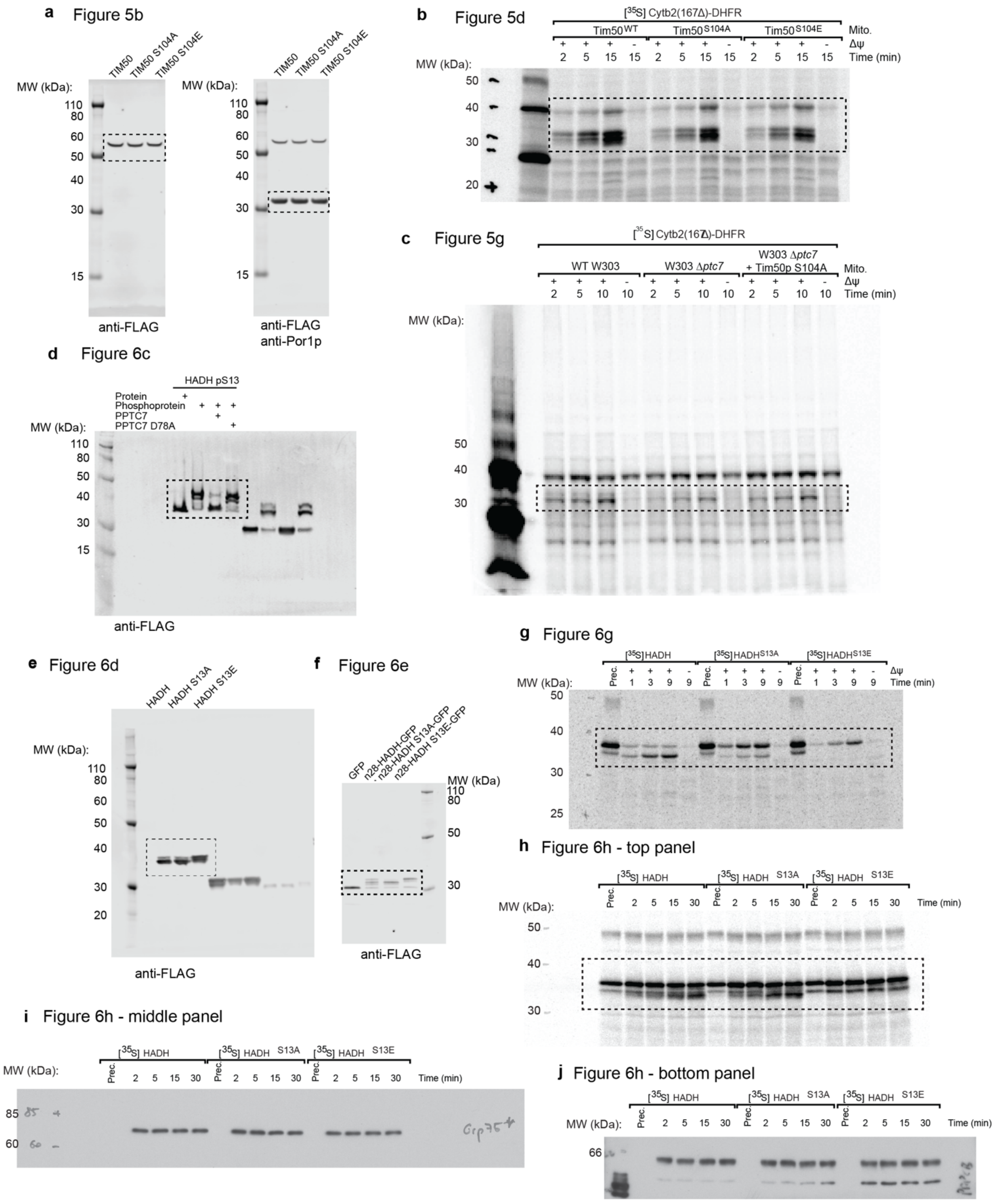

Supplementary Figure 7: Uncropped and unprocessed Western blot and autoradiography images from the main text. Associated figures in the main text are identified in the figure. Dotted boxes represent cropped regions shown in manuscript. Westerns are identified by their associated antibodies beneath unprocessed blots; if no antibody is listed, the gel represents an autoradiograph. Further details regarding blots can be found in the main figure legends.

SUPPLEMENTARY TABLE

Supplementary Table 1: Primers used in this study.

**Oligo name**	**Sequence**	**Use**	**Figure**
Pptc7 exon 2 target seq.	CTTTAATGCGAACATGCGAG	CRISPR design	1a
Pptc7 exon 3 target seq.	CCGCTTGCACACAGCGAACC	CRISPR design	1a
Pptc7 exon 2 targeting CRISPR oligo	gaaattaatacgactcactataggCTTTAATGCGAACATGC GAGgttttagagctagaaatagc	Pptc7 exon 2 - CRISPR	n/a
Pptc7 exon 3 targeting CRISPR oligo	GaaattaatacgactcactataggCCGCTTGCACACAGCGA ACCgttttagagctagaaatagc	Pptc7 exon 3 - CRISPR	n/a
Chi Long R	Aaaagcaccgactcggtgccactttttcaagttgataacggactagccttat tttaacttgctatttctagctctaaaac	oligo - sgRNA generation	n/a
Pptc7 geno exon 2 fwd	AGCAGATGATTTGGCAGTGTGGA	Genotyping Pptc7 exon 2	S1a
Pptc7 geno exon 2 rev	CCAGAAAGGATCTGGCAATGACAA	Genotyping Pptc7 exon 2	S1a
Pptc7 geno exon 3 fwd	TAGTAAGACCCAGTAACCTGCTGT	Genotyping Pptc7 exon 3	S1a
Pptc7 geno exon 3 rev	ACTAACTGGTCCTGAGGACGTCA	Genotyping Pptc7 exon 3	S1a
Pptc7 NGS exon 2 fwd	ACACTCTTTCCCTACACGACGCTCTTCC GATCTTTCTCAGGGACTTTAATGCGAACATGC	ex. 2 amplicon for NGS	1e; S1i-t
Pptc7 NGS exon 2 rev	GTGACTGGAGTTCAGACGTGTGCTCTTC CGATCTTCTGGTTTGGTTGGCTATCAG	ex. 2 amplicon for NGS	1e; S1i-t
Pptc7 NGS exon 3 fwd	ACACTCTTTCCCTACACGACGCTCTTCCG ATCTGGATGCCCATGTTGTTTGTAGG	ex. 3 amplicon for NGS	1e; S1i-t
Pptc7 NGS exon 3 rev	GTGACTGGAGTTCAGACGTGTGCTCTTC CGATCTGTGCTGCTGCTCGTCAGACC	ex. 3 amplicon for NGS	1e; S1i-t
Ndufa9 qPCR fwd	GGAGGACAGGTTCCTTAATCAC	qPCR	3e
Ndufa9 qPCR rev	CCTTGGAAACATCTGCAACATAC	qPCR	3e
Uqcrc2 qPCR fwd	CTTCTACCGTCCTTCAACAGATT	qPCR	3e
Uqcrc2 qPCR rev	AGGCGTATGTCCCAAGTTTC	qPCR	3e
Cox4 qPCR fwd	AGTTGTACCGCATCCAGTTT	qPCR	3e
Cox4 qPCR rev	GGCCATACACATAGCTCTTCTC	qPCR	3e
Atp5a1 qPCR fwd	CGTCTGACCGAGTTGCTAAA	qPCR	3e
Atp5a1 qPCR rev	GGGCTCCAGTTTGTCAAGATA	qPCR	3e
Cs qPCR fwd	GTGACCATGAGGGTGGTAATG	qPCR	3e
Cs qPCR rev	TGCTGCAAAGGACAGGTAAG	qPCR	3e
Sdha qPCR fwd	CACACGTCTACCCGGAATTT	qPCR	3e
Sdha qPCR rev	CACCTGTCCCTTGTAGTTAGTG	qPCR	3e
Cpt1a qPCR fwd	CCATGAAGCCCTCAAACAGATC	qPCR	3e
Cpt1a qPCR rev	ATCACACCCACCACCACGATA	qPCR	3e
Pptc7 qPCR fwd	ACAGTTTGCATGTGACAATGG	qPCR	3e
Pptc7 qPCR rev	CCGTGTACTCAGCCACTATTG	qPCR	3e

SUPPLEMENTARY METHODS

Selection of mice for experimental procedures

Most studies were performed with P0 mouse pups sacrificed within 24 hours of their birth unless otherwise noted. Sex was not determined for most experiments analyzing perinatal pups as males and females are indistinguishable at P0. Whole litters were separated from their mothers, weighed, examined for morphological differences, and sacrificed via decapitation. Tail tips were simultaneously collected from each pup for genotyping purposes. Littermates were typically selected according to genotype and randomly assigned to experimental groups. Studies performed with P1 or P2 mice used the same workflow as above, except mice were sacrificed 1 or 2 days after birth, respectively. Dead pups found in cages were genotyped using tissue isolated from their carcasses, but these tissues/samples were not used in any downstream experiments.

Genotyping strategy

Tail tips were isolated from each mouse and used as a source of genomic DNA (gDNA). Tails were resuspended in 600 µl of Genomic Lysis Solution (20 mM Tris, pH 8.0, 150 mM NaCl, 100 mM EDTA, 1% SDS) supplemented with 3 µl concentrated proteinase K (19.5 mg/ml, Roche #03115887001) and incubated at 55ºC overnight. After the overnight incubation, samples were retrieved and allowed to cool to room temperature for 10 min. before the addition of 200 µl Protein Precipitation Solution (Qiagen #158910). Samples were then incubated on ice for 5 min. before vortexing for ~15 s. per sample. Samples were centrifuged (16K x g) for 5 min. to pellet precipitated proteins. The supernatant was removed and supplemented with 600 µl of 100% isopropanol, which was inverted ~25x to precipitate DNA. Precipitated DNA was pelleted (16K x g, 3 min.) and liquid aspirated. The pellet was washed with 600 µl 70% ethanol and subjected to a final spin (16K x g, 3 min.). The liquid was again aspirated, the pellet allowed to dry for ~15-30 min. at room temperature, and the gDNA hydrated with ~200 µl of ultrapure water. gDNA was quantified using a Nanodrop (Thermo-Fisher) and ~300 ng of gDNA was added to each genotyping reaction.

The indels formed in the Pptc7 model were too small to be tracked by size differences (1 bp and 4 bp deletions), so we created a genotyping strategy that exploited unique restriction sites that were either created (E3d1) or destroyed (E2d4) within the indel. Within exon 2, the 4 bp deletion disrupted a naturally-occurring BsrBI site (CCG_^CTC; see MGI:6094244 and Figure S1A for allele information). This enabled amplification of an ~600 bp fragment flanking the indel using primers 5'- AGCAGATGATTTGGCAGTGTGGA-3' (forward) and 5'-CCAGAAAGGATCTGGCAATGACAA-3' (reverse). This fragment was cut with BsrBI (NEB #R0102) for 60 min. at 37°C, resulting in wild type alleles that digested to ~ 320 + 280 bp fragments and E2d4/KO alleles that did not digest (resulting in a full length ~600 bp fragment). Within exon 3, the 1 bp deletion generated a PasI site (CC^CWGGG; see MGI:6143811and Figure S1A for allele information). To genotype this region, a 330 bp fragment flanking the indel was amplified using primers 5'-TAGTAAGACCCAGTAACCTGCTGT-3' (forward) and 5'-ACTAACTGGTCCTGAGGACGTCA-3' (reverse). This fragment was digested with PasI (Thermo-Fisher #ER1861) for 60 min. at 55°C, resulting in wild type alleles that did not digest (330 bp) and E3d1/KO alleles that digested to ~75 + 255 bp. After restriction digest, samples were run on a 1% agarose gel (TAE) and distinct band patterns were used to distinguish WT and KO alleles as described above. Primers used for amplification of alleles can be found in Supplementary Table 1.

Next generation sequencing

Tissue and gDNA isolation: The F0 founder mouse was sacrificed at 18 months of age via CO_2 asphyxiation. To test for mosaicism, a subset of its tissues was isolated and snap frozen in liquid nitrogen until gDNA extraction. Tissues harvested include brain (cerebrum), small intestine, kidney, skeletal muscle (gastrocnemius), spleen, stomach, liver, and heart. In parallel, two heterozygous F1 offspring (age 15 months, female) were sacrificed and liver and heart tissues isolated and processed as described above. These F1 offspring were genotype verified as carrying either an exon 2 deletion or an exon 3 deletion, and thus served as internal controls for both allelic variants, as well as wild type alleles at their non-targeted locus (i.e. E2 heterozygous mouse served as a wild type control at E3 and vice versa). Genomic DNA (gDNA) was harvested from each tissue using a Qiagen DNeasy Blood & Tissue Kit (Qiagen #69504) according to the manufacturer's protocol.

Target amplification and initial index PCR: Each sample was amplified using genotyping primers for E2 or E3 as a template for NGS-compatible sequencing (see Supplementary Table 1 for primers). From these amplicons, index PCR fragments were generated using i5/i7 compatible NGS primers (see Supplementary Table 1 for sequences). Amplicons were verified by visualizing 10% of the reaction via gel electrophoresis, and DNA quality was verified through A260/280 ratios between 1.8-2.0. The remaining PCR reactions were purified using a GeneJet PCR purification kit (Thermo-Fisher #K0702) according to manufacturer's instructions.

Construction and sequencing of indexed libraries from custom amplicons: Purified amplicons were submitted to the University of Wisconsin-Madison Biotechnology Center. Region specific amplicon primers were designed to contain the following 5' regions appropriate for nested PCR (Sequences can be found in Supplementary Table S1). Libraries were prepared with guidance from Illumina's 16s Metagenomic Sequencing Library Preparation Protocol, Part #15044223 Rev. B (Illumina Inc., San Diego, California, USA), with slight modifications. Illumina dual indexes and Sequencing adapters were added with sequences are equivalent to the Illumina Dual Index adapters D501-D508 and D701-D712). Following PCR, libraries were cleaned using a 0.9x volume of AxyPrep Mag PCR clean-up beads (Axygen Biosciences). Quality and quantity of the finished libraries were assessed using an Agilent DNA1000 chip and Qubit® dsDNA HS Assay Kit, respectively. Libraries were standardized to 2nM and pooled prior to sequencing. Paired end, 150 bp sequencing was performed using the Illumina MiSeq Sequencer and a MiSeq 300 bp (v2) sequencing cartridge. Images were analyzed using the standard Illumina Pipeline, version 1.8.2.

NGS Data analysis: Analysis of NGS data was performed by the University of Wisconsin (UW) biotechnology center. Briefly, sequencing reads were adapter and quality trimmed using the Skewer[1] trimming program. Flash[2] was used to merge paired end reads into amplicon sequences. Amplicons were then aligned to the reference genome, {GENOME}, using BWA-MEM[3], and preformed local realignment using GATK[4]. Variants were called using GATK[5]. HaplotypeCaller version 3.6 and annotated with SNPeff[5]. From these analyses, a total of 8000 reads were used for each tissue at each indel to generate the percentage allele frequency shown in the figures.

Metabolomics and Lipidomics

Tissue preparation: Heart and liver tissues were immediately harvested and snap frozen in liquid nitrogen unless otherwise noted. Tail tips were simultaneously collected from each pup for genotyping purposes. Liver and heart tissues were cryo-pulverized with mortar and pestle in liquid nitrogen. Approximately 17 mg of frozen tissue was transferred to new microcentrifuge tubes,

samples were extracted with 4°C 7:2:1 HPLC-grade methanol:water:chloroform at an extraction ratio of 1 mL solvent/30 mg tissue. After addition of extraction solvent, samples were immediately probe sonicated on ice for 10 sec., incubated at 4°C for 5 min, then centrifuged at 10000 x g for 10 min at 4°C to pellet precipitated protein. A portion of the extract was aliquoted into glass autosampler vials and dried by vacuum centrifugation (150 µL for gas chromatography analysis, 100 µL for liquid chromatography analysis). To obtain more hydrophobic lipids, additional chloroform was added to a final of 50% of total volume; samples were sonicated again for 10 sec., centrifuged at 10000 x g for 10 min. The extract was aliquoted in glass autosampler vials and dried by vacuum centrifugation.

GC-MS metabolomics: For GC-MS analysis, dried extract was derivatized for 90 min. with 20 mg/mL methoxyamine hydrochloride in pyridine at 20°C (25 µL) and then with MSTFA for 30 min at 37°C (25 µL). Samples were analyzed by GC-Orbitrap; 1 µL of sample, split 1:10, was injected onto a TraceGOLD TG-5SilMS GC column (cat. no. 26096-1420, Thermo Scientific). Temperature was held at 50°C for 1 min., then ramped to 320°C at rate of 11°C/min., then held at 320°C for 4.40 min. Molecules were analyzed with positive electron-impact (EI)-Orbitrap full scan of 50-650 m/z range. Raw files were analyzed using an in-house tool for deconvolution, retention-time alignment, and peak quantitation, see Stefely et. al, 2016[6]. Deconvolved peaks were searched against NIST 2014[7] and in-house high-resolution GC libraries; retention index was used to filter search hits. A single quant-ion was used for quantification of deconvolved peaks. Quantified peaks in samples were included if they were at least 10-fold greater than peaks quantified in solvent blanks.

Acylcarnitine analysis: For acylcarnitine analysis, dried samples were resuspended in 50 µL of solvent containing 98% Mobile Phase A (5 mM Ammonium acetate, pH 6.8) and 2% Mobile phase B (5 mM Ammonium acetate, 95% acetonitrile, pH 6.8). Samples were separated on a Water's Acquity UPLC CSH C18 Column (2.1 mm x 100 mm) with a 5 mm VanGuard Pre-Column using the following 14 min gradient: 2% B for 1 min (0.4 mL/min), increased to 95% B over next 7 min (0.4 mL/min), held at 95% B for 2 min, decreased to 2%B over next 1 min, then equilibrated at 2% B for 3 min. Samples were analyzed with positive ion mode electrospray ionization (ESI) with full scan MS1 (150-1000 Th) collected 17,000 resolving power (at 400 m/z) for 0-14 min and top-2 data dependent MS2 scans fragmented with stepped normalized collision energy (20-40%). Aclycarnitines were quantified using Thermo's Tracefinder application.

CoQ measurements: For lipid analysis, dried extract was resuspended in 65:30:5 isopropanol:acetonitrile:water. Lipid LC-MS analysis was performed on a Water's Acquity UPLC CSH C18 Column (2.1 mm x 100 mm) with a 5 mm VanGuard Pre-Column Mobile coupled to a Q Exactive Focus. Mobile phase A consisted of 70% acetonitrile and 30% water with 10 mM Ammonium acetate and 0.025% acetic acid. Mobile phase B consisted of 90% isopropanol and 10% acetonitrile with 10 mM ammonium acetate and 0.025% acetic acid. Samples were separated using the following 30 min gradient: 2% B for 2 min (0.4 mL/min), increased to 30% B over next 3 min (0.4 mL/min), increased to 50% B over next 1 min (0.4 mL/min), increased to 85% B over next 14 min (0.4 mL/min), increased to 99% B over next 1 min and held at 99% B for 7 min (0.3 mL/min); then returned to 2% to equilibrate for 2 min (0.4 mL/min). Samples were analyzed with alternating positive and negative ion mode electrospray ionization (ESI) with full scan MS1 (150-1600 Th) collected 17,000 resolving power (at 400 m/z) for 0-30 min and top-2 data dependent MS2 scans fragmented with stepped normalized collision energy (20-40%). Co-enzyme Q intermediates were detected with parallel reaction monitoring (PRM) mode: CoQ6 (591.4408 m/z, 8.0-11.0 min), CoQ8 (727.5660 m/z, 11.0-13.2 min), CoQ9 (795.6286 m/z, 13.2-14.7 min), and

CoQ10 (880.71820 m/z, 14.7-17.0 min). Raw files were quantified using the Thermo Compound DiscovererTM 2.0 application with peak detection, retention time alignment, and gap filling. Only peaks 10-fold greater than solvent blanks were included in the later analysis. Identification was aided by in-house software[8] and lipid libraries; only compounds with definitive MS/MS evidence were assigned with identification. CoQ intermediates were quantified using Thermo's Tracefinder application.

Enzyme assays

Citrate synthase activity assay: Citrate synthase activity was assayed as previously described[9]. Briefly, genotype verified tissues were thawed (n=3-4 each genotype, wild type and *Pptc7* knockout heart and tissues) and resuspended in Lysis Buffer A (LBA) (50 mM Tris, pH 7.4, 40 mM NaCl, 1 mM EDTA, 0.5% triton-X 100) supplemented with 1x protease (500 μg/ml final concentration of each of the following inhibitors: Pepstatin A, Chymostatin, Antipain, Leupeptin, Aprotinin) and phosphatase (50 mM NaF, 10 mM β-glycerolphosphate) inhibitors. Lysates were quantified using the Bicinchoninic acid (BCA) assay (Pierce #23225). 5 μg of total protein lysate was added to a 190 μl reaction containing 100 mM Tris, pH 7.4, 300 μM acetyl-CoA, and 100 μM 5,5'- dithio-bis(2-nitrobenzoic acid) (DTNB). Reactions were started by addition of oxaloacetate (10 μl to a final [c] of 500 μM) and were monitored by DTNB absorbance (A412) on a Cytation 3 plate reader (BioTek). Relative rates were calculated using the maximum velocity function on the BioTek Gen5 analysis software suite, and rates were normalized to the average of 4x samples of wild type citrate synthase activity. Error was calculated using standard deviation, and significance was calculated using a Student's t test.

HADH activity assay: Hadh activity was assayed as previously described[10]. Briefly, recombinant human HADH (Entrez Gene #3033) or HADH site-specifically phosphorylated at S13 (see "Phosphoserine incorporation of recombinant proteins using cell free protein synthesis" for more details) were generated with a C-terminal FLAG tag using cell free protein synthesis. 50 μl reactions of HADH and HADH pS13 were brought up to 500 ul total volume in Lysis Buffer A (LBA) (50 mM Tris, pH 7.4, 40 mM NaCl, 1 mM EDTA, 0.5% triton-X 100) supplemented with 1x protease (500 μg/ml final concentration of each of the following inhibitors: Pepstatin A, Chymostatin, Antipain, Leupeptin, Aprotinin) and phosphatase (50 mM NaF, 10 mM β-glycerolphosphate) inhibitors. Proteins were IPed using M2-FLAG antibody-conjugated magnetic beads with nutation for 1 hour at 4°C. IPed proteins were washed 3 times with LBA and eluted in 50 μl FLAG peptide in TBS at a final concentration of 200 μg/μl for 30 minutes at room temperature with constant shaking. HADH activity was assayed using 2.5 μl eluate from each IP (corresponding to ~200 ng recombinant protein per reaction), and IPed proteins were run on an SDS-PAGE gel and Western blotted for FLAG to verify equal protein content. HADH activity assays were run in 100 μl reactions containing 100 mM Tris, pH 7.4, 1 mM EDTA, 500 μM acetoacetyl-CoA, and 250 μM NADH (all components listed at final concentrations). All reaction components were placed in a master mix except substrate (acetoacetyl-CoA), which was added to initiate the reaction. Reactions were monitored by decreases in A340, which corresponds to the oxidation of NADH, on a Cytation 3 plate reader (BioTek). Relative rates were calculated using the maximum velocity function on the BioTek Gen5 analysis software suite, and rates were normalized to the average of 4x samples of wild type HADH activity. Error was calculated using standard deviation, and significance was calculated using a Student's t test.

Quantitative multiplexed proteomics and phosphoproteomics

Protein Digestion and Isolation: Protein from lysed, homogenized mouse tissues was digested into tryptic peptides following previously reported protocols[11]. Each tissue sample was suspended in 500 µL guanadinium chloride (6M) and homogenized with a cup horn sonicator (Qsonica) until fully in solution. Protein was precipitated in 5 mL methanol and pelleted (5,000xG, 15 min). Pellet was resuspended in 500 uL lysis buffer (8M urea, 100 mM tris pH 8, 10 mM TCEP, 40 mM 2-chloroacetamide). Trypsin was added (1:50::g:g) and incubated overnight at ambient temperature with gentle rocking. Ratios were based off of expected yields from previous experiments (Liver – 2 mg, Heart – 0.8 mg). An addition bolus of trypsin (1:200:g:g) was added in the morning. Solution was incubated for an additional hour before peptides were isolated and desalted with StrataX polymeric reverse phase resin (Phenomenex). Peptide yield was calculated by bicinchoninic acid (BCA) assay (Thermo Pierce).

TMT Labelling: 0.35 mg of each heart sample and 0.5 mg of each liver sample were suspended in 100 µL triethylammonium bicarbonate (TEAB, 200 mM) and combined with 0.8 mg TMT label in 50 µL acetonitrile. Solutions were shaken for 6H at ambient temperature before quenching with 0.8 µL 50% hydroxylamine. 2 µL of each sample for each 10-plex experiment was combined and analyzed for labelling efficiency (see results). Samples were concentrated without needing to correct for labelling efficiency.

Phosphopeptide Enrichment: Phosphopeptide enrichment with titanium chelation (Ti-IMAC, ReSyn Biosciences) was performed on pooled samples using previously reported methods[12]. All samples from each 10-plex experiment were combined in 1 mL 80% acetonitrile (ACN) + 6% trifluoroacetic acid (TFA). 420 µL and 600 µL of magnetic beads (Mag-ReSyn - Ti-IMAC, Resyn Biosciences) for heart and liver, respectively, were washed 3X with 1 mL 80% ACN + 6% TFA before combining with peptide sample. Samples were shaken at ambient temperature for 20 min. Supernatant was separated from bead and kept as the "unenriched" sample. Beads were washed 3X with 1 mL 80% ACN + 6% TFA, 1X with 1 mL 80 % ACN, 1X with 1mL 80% ACN + 0.5 M glycolic acids, and 1X with 1 mL 80% ACN before eluting with 2X 300 µL 50% ACN + 1% ammonium hydroxide. Eluent was concentrated to dryness, resuspended in 0.2% formic acid, desalted, and once more, concentrated to dryness.

High pH Reverse Phase Fractionation: 500 µg of each unenriched sample and the entirety of each enriched sample were separated over an Acquity BEH C18 reverse phase column held at 60 °C (130 Å pore size, 1.7 µm particle size, 2.1 x 100 mm, Waters Corp) using a Dionex Ultimate 3000 uHPLC (600 µL/min flow rate, Thermo Scientific). Mobile phase A consisted of 20 mM ammonium bicarbonate in H2O. Mobile phase B consisted of 20 mM ammonium bicarbonate in ACN/H2O. Elution was achieved with the gradient: 0 min, 0%B; 2 min, 0%B; 5 min, 15%B; 20 min, 50%B; 23 min, 100%B; 25 min, 100%B; and 25.5 min, 0%B. 10x 1 min. fractions were collected from 3-23 minutes with concatenation of fraction 1 and 11, 2 and 12, 10 and 20.

LC-MS/MS Analysis: Concentrated fractions of enriched and unenriched peptides were resuspended in 20 µL and 50 µL 0.2% formic acid, respectively. 4 µL and 2 µL of each enriched and unenriched sample, respectively, were analyzed on a Q-LTQ-OT mass spectrometer (Thermo Scientific Fusion Lumos) following LC separation. Enriched samples were run in duplicate. Injections were made onto a 75-360 µm inner-out diameter fused silica capillary column with a laser-pulled electrospray tip packed with BEH C18 (130 Å pore, 1.7 µm particle size, 35 cm, Waters Corp) with a Dionix Ultimate 3000 uHPLC. Peptides were separated using the following gradient: 0 min, 0%B; 14 min, 5%B; 75 min, 55%B; 76 min, 100%B; 79 min, 100%B; 80 min, 0%B; 90 min, 0%B. Eluting peptides entered into the mass spectrometer following positive mode electrospray ionization. MS1 survey scans were performed in the orbitrap (60K resolution, AGC

target – 1e6, 50 ms max injection time). MS2 analysis of HCD-generated (35% NCI) fragment ions were also performed in the orbitrap (60K resolution, AGC target – 2e5, 118 ms max injection time). Monoisotopic precursor selection and dynamic exclusion (60 s) were enabled.

Data Analysis: Thermo RAW files were searched with the Open Mass Spectrometry Search Algorithm (OMSSA) within the Coon OMSSA Proteomic Analysis Software Suite (COMPASS) against a database of canonical proteins and isoforms (Uniprot, *Mus musculus*)[13,14]. OMSSA-searchable DTA files were generated from Thermo RAW files and OMSSA searching was performed on the University of Wisconsin-Madison Condor server. Searches were performed with precursor mass tolerance of 150 ppm and product ion mass tolerance of 0.015 Da. N-terminal and lysine TMT modifications were imposed as fixed modifications. TMT modification of tyrosine was used as a variable modification. Additionally, phosphorylation of serine, threonine, and tyrosine, as well as neutral losses, were set as variable modifications for enriched samples. Sites of phosphorylation were considered localized if given a localization score >0.75 by the PhosphoRS module within COMPASS[15]. Search results were filtered to a peptide false discovery rate of 1%. Proteins from unenriched samples were also filtered to a final false discovery rate of 1%. Phosphopeptides from enriched samples were localized with the PhosphoRS feature within COMPASS. Phosphopeptide intensities were normalized to the total reporter ion intensity at the protein level. Also available are phosphopeptide intensities normalized to protein mean-normalized fold change across samples. For each localized phosphopeptide, a mean fold change was calculated by taking the difference of averaged, log2 transformed intensities from each condition. An associated P value was calculated using Student's t test assuming equal variance. Multiple hypothesis testing was performed by Benjamini-Hochberg correction.

Cloning and site directed mutagenesis

Site directed mutagenesis was performed on mammalian, bacterial, or cell free expression vectors. Briefly, plasmid templates were normalized to 25 ng/μl and 0.5 μl was used in a 25 μl reaction containing: 2.5 μl 10x PfuUltra DNA polymerase buffer (Agilent), 0.75 μl DMSO, 0.5 μl PfuUltra DNA polymerase, 0.5 μl plasmid template, and 5 μl 1 μM forward and reverse SDM primers, and water. After PCR, reactions were digested with DpnI for 1 hour before transformation into DH5α cells and plating on LB-plates containing appropriate antibiotics. Colonies were selected, miniprepped, and verified via Sanger sequencing before use in downstream applications.

Mammalian cell culture

293 cells were cultured in DMEM (high glucose, no pyruvate, Thermo-Fisher catalog #11965-092) supplemented with 10% heat inactivated fetal bovine serum (FBS) and 1x penicillin/streptomycin. Cells were grown in a temperature-controlled CO_2 incubator at 37°C and 5% CO_2. Cells were subcultured using 0.25% trypsin-EDTA every 2-3 days. For transfections, cells were split to ~40% confluence on Day 1. On Day 2, cells were transfected with 7.5 μg Maxiprep-purified plasmid supplemented with 20 μg linear polyethylenimine (PEI, PolySciences), and 900 μL Opti-MEM (Life Technologies) as previously described[16].

Yeast strains used

Various strains derived from the haploid W303 (*his3 leu2 lys2 met15 trp1 ura3 ade2*) yeast were used for mitochondrial isolation for import assays. A *ptc7Δ* strain was generated in W303 yeast (*his3 leu2 lys2 met15 trp1 ura3 ade2 ptc7*:*KanMX6*) as previously described[9] through replacement

of the ptc7 open reading frame with the Kan3MX6 cassette. The Tim50p studies were performed with a previously described W303 strain in which the *TIM50* ORF was placed under control of the *GAL7* promoter[17]. The Tim50p studies were performed with YPH499 (*ura3-52 lys2-801_amber ade2-101_ochre trp1-Δ63 his3-Δ200 leu2-Δ1*) yeast strains expressing Tim50 S104E and S104A mutations, which were generated by plasmid shuffling as described previously[18].

Mitochondrial import assays

Isolation of mitochondria from yeast: For mitochondrial isolation from yeast, a single colony of yeast was incubated in 3-4 ml of YPD (yeast extract (10 g/L), peptone (20 g/L), and dextrose (20 g/L)) media overnight. 1 x 10^8 cells from this culture were diluted into 500 ml of YEP (yeast extract (10 g/L), peptone (20 g/L)) media supplemented with sterile-filtered glycerol (final [c] = 3% v/v) and dextrose (final [c] = 0.1% v/v). Yeast were grown at 30°C in an orbital shaker (230 rpm) for 19-20 hrs to a final OD of ~5-6 (with an OD of 1 = 1e7 cells). Mitochondria were isolated as previously described for mitochondrial import[19]. Briefly, cells were collected by centrifugation at 3000 x g for 5 min. at room temperature, and wet weight of the cells was determined. The yeast pellet was resuspended in 2 mL/g prewarmed DTT buffer (100 mM Tris-H_2S0_4, pH 9.4, 10 mM dithiothreitol (DTT)) and shaken slowly (80 rpm) at 30°C for 20 min. After this, cells were again pelleted (3000 x g, 5 min, room temp) and washed in Zymolyase buffer (1.2M sorbitol, 20 mM potassium phosphate, pH 7.4). Cells were again pelleted and resuspended in Zymolyase buffer containing 3 mg Zymolyase per gram wet weight of yeast, and yeast were shaken slowly at 30°C for 45 min. Cells were again pelleted and spheroplasts were resuspended in ice-cold homogenization buffer (0.6M sorbitol, 10 mM Tris, pH 7.4, 1 mM ETDA, 1 mM PMSF, 0.2% essentially fatty acid free BSA) and homogenized using 15 strokes of a tight-fitting glass-Teflon homogenizer. The homogenate was spun (1500 x g, 5 min, 4°C) to pellet cell debris and nuclei; the supernatant was spun again at low speed (4000 x g, 5 min, 4°C) to pellet remaining debris, and transferred to a fresh centrifuge tube. The supernatant was spun at 12000 x g for 15 min at 4°C to pellet mitochondria. Isolated mitochondria were resuspended using wide-bore tips to a final concentration of 10 mg/ml in SEM buffer (250 mM sucrose, 1 mM EDTA, and 10 mM MOPS, pH 7.2), snap frozen in liquid nitrogen, and stored at -80°C until use in import assays.

Generation of 35S labeled precursors: Recombinant HADH or cytochrome b2-$(167)_{\Delta 19}$-DHFR (a gift from Elizabeth Craig) were generated using the quick TnT® Quick Coupled Transcription/Translation System (Promega) using either the T7 kit (HADH) or the SP6 kit (cytb2-DHFR) according to the manufacturer's instructions. Briefly, 40 µl of reticulocyte lysate was supplemented with 1 µg maxiprepped DNA, 20 µCi 35S-EasyTag EXPRESS35S protein labeling mix (Perkin Elmer), and supplemented with water to a 40 µl final volume. Reactions were incubated for 90 minutes in a thermal block set to 30°C. Reactions were terminated by placing reactions on ice until use in mitochondrial import assays.

Isolation of mitochondrial from 293T cells: For mammalian targets, radiolabeled HADH (WT, S13A, S13E) precursor proteins were generated by in vitro transcription/translation reactions in rabbit reticulocyte lysate in the presence of ^{35}S-Methionine. Mitochondria were isolated from HEK293T cells as described before (Johnston et al. JBC 2002) and resuspended in import buffer (250 mM sucrose, 5 mM magnesium acetate, 80 mM potassium acetate, 10 mM sodium succinate, 20 mM HEPES-KOH, pH 7.4) supplemented with 1 mM DTT and 5 mM ATP.

Phosphoserine incorporation in recombinant proteins using cell free protein synthesis

Generation of bacterial lysate: The bacterial strain C321.ΔA.Δserb.AmpS was a gift from Jesse Rinehart (Addgene plasmid #68306) and was used to generate lysates for the CFPS reactions. The bacteria were transformed with SepOTSλ, a gift from Jesse Rinehart (Addgene plasmid #68292) to allow for generation of phosphoserine-incorporated recombinant proteins to be generated. *E. coli* C321.ΔA cells harboring the Sep-OTS system[20] were grown in 2 × YTPG media supplemented with 2 mM L-phosphoserine at 30 °C. The SepOTS system was induced at an OD600 of 0.6 with 1 mM isopropyl-β-D-thiogalactoside (IPTG) and cells were grown to a final OD600 of 3.0 that represents the middle of the exponential growth phase. Cultures were then collected on ice and cells were pelleted by centrifugation performed for 15 min at 5,000 x *g* and 4 °C. Cell pellets were then washed three times with cold S30 buffer (10 mM Tris-acetate pH 8.2, 14 mM magnesium acetate, 60 mM potassium acetate and 1 mM dithiothreitol). After final wash and centrifugation, the pelleted wet cells were weighed, flash frozen in liquid nitrogen and stored at −80°C. To make cell extract, the thawed cells were suspended in 0.8 ml of S30 buffer per 1 g of wet cell mass and processed as reported by Kwon and Jewett[21].

CFPS reactions: Each 30 µl cell free protein synthesis reaction was set up with 15 µl of cell free master mix (1.2 mM ATP, 0.85 mM each of GTP, UTP, and CTP (pH 7 – 7.2); 34 µg/mL folic acid; 170 µg/mL of *E. coli* tRNA mixture; 2 mM each of standard amino acids (omitting methionine and cysteine in S35 experiments); 0.33 mM NAD; 0.27 mM coenzyme A; 1.5 mM spermidine; 1 mM putrescine; 4 mM oxalic acid; 130 mM potassium glutamate; 10 mM ammonium glutamate; 12 mM magnesium glutamate; 33 mM phosphoenolpyruvate (pH 7), 2 mM phosphoserine, 57 mM HEPES pH 7), 100 µg/mL T7 RNA polymerase, 9 µl of processed C321.ΔA.Δserb.AmpS E. coli lysate (see above for details), and 13.3 ng/µl final [c] maxiprepped plasmid DNA encoding the ORF of the protein to be transcribed and translated, including a TAG at the position at which a phosphoserine residue is to be incorporated. Reactions were mixed thoroughly and incubated in a thermocycler held at 30°C for 20 hours.

SUPPLEMENTARY REFERENCES

1. Jiang, H., Lei, R., Ding, S.W. & Zhu, S. Skewer: a fast and accurate adapter trimmer for next-generation sequencing paired-end reads. *BMC Bioinformatics* **15**, 182 (2014).
2. Magoc, T. & Salzberg, S.L. FLASH: fast length adjustment of short reads to improve genome assemblies. *Bioinformatics* **27**, 2957-63 (2011).
3. Li, H. Aligning sequence reads, clone sequences and assembly contigs with BWA-MEM. *arXiv* **1303.3997**(2013).
4. McKenna, A. *et al.* The Genome Analysis Toolkit: a MapReduce framework for analyzing next-generation DNA sequencing data. *Genome Res* **20**, 1297-303 (2010).
5. Cingolani, P. *et al.* A program for annotating and predicting the effects of single nucleotide polymorphisms, SnpEff: SNPs in the genome of Drosophila melanogaster strain w1118; iso-2; iso-3. *Fly (Austin)* **6**, 80-92 (2012).
6. Stefely, J.A. *et al.* Mitochondrial protein functions elucidated by multi-omic mass spectrometry profiling. *Nat Biotechnol* **34**, 1191-1197 (2016).
7. Linstrom, J. NIST Standard Reference Database Number 69. (2014).
8. Hutchins, P.D., Russell, J.D. & Coon, J.J. LipiDex: An Integrated Software Package for High-Confidence Lipid Identification. *Cell Syst* **6**, 621-625 e5 (2018).
9. Guo, X. *et al.* Ptc7p Dephosphorylates Select Mitochondrial Proteins to Enhance Metabolic Function. *Cell Rep* **18**, 307-313 (2017).
10. Fernandez, M., Mano, S., de Fernando, D.G., Ordonez, J.A. & Hoz, L. Use of beta-hydroxyacyl-CoA-dehydrogenase (HADH) activity to differentiate frozen from unfrozen fish and shellfish. *European Food Research and Technology* **209**, 205-208 (1999).
11. Hebert, A.S. *et al.* The one hour yeast proteome. *Mol Cell Proteomics* **13**, 339-47 (2014).
12. Riley, N.M. *et al.* Phosphoproteomics with Activated Ion Electron Transfer Dissociation. *Anal Chem* **89**, 6367-6376 (2017).
13. Geer, L.Y. *et al.* Open mass spectrometry search algorithm. *J Proteome Res* **3**, 958-64 (2004).
14. Wenger, C.D., Phanstiel, D.H., Lee, M.V., Bailey, D.J. & Coon, J.J. COMPASS: a suite of pre- and post-search proteomics software tools for OMSSA. *Proteomics* **11**, 1064-74 (2011).
15. Taus, T. *et al.* Universal and confident phosphorylation site localization using phosphoRS. *J Proteome Res* **10**, 5354-62 (2011).
16. Floyd, B.J. *et al.* Mitochondrial Protein Interaction Mapping Identifies Regulators of Respiratory Chain Function. *Mol Cell* **63**, 621-632 (2016).
17. Yamamoto, H. *et al.* Tim50 is a subunit of the TIM23 complex that links protein translocation across the outer and inner mitochondrial membranes. *Cell* **111**, 519-28 (2002).
18. Chacinska, A. *et al.* Mitochondrial presequence translocase: switching between TOM tethering and motor recruitment involves Tim21 and Tim17. *Cell* **120**, 817-29 (2005).
19. Meisinger, C., Pfanner, N. & Truscott, K.N. Isolation of yeast mitochondria. *Methods Mol Biol* **313**, 33-9 (2006).
20. Pirman, N.L. *et al.* A flexible codon in genomically recoded Escherichia coli permits programmable protein phosphorylation. *Nat Commun* **6**, 8130 (2015).
21. Kwon, Y.C. & Jewett, M.C. High-throughput preparation methods of crude extract for robust cell-free protein synthesis. *Sci Rep* **5**, 8663 (2015).
