# Supplementary material for: Pptc7 is an essential phosphatase for promoting mammalian mitochondrial metabolism and biogenesis
Source: Nat Commun. 2019 Jul 19;10:3197. doi: 10.1038/s41467-019-11047-6 (PMC6642090; doi:10.1038/s41467-019-11047-6)
Supplement: Supplementary file 5 — Reporting Summary [file 41467_2019_11047_MOESM5_ESM.pdf]

nature research

Corresponding author(s): David J. Pagliarini

Last updated by author(s): June 3, 2019

Reporting Summary

Nature Research wishes to improve the reproducibility of the work that we publish. This form provides structure for consistency and transparency in reporting. For further information on Nature Research policies, see Authors & Referees and the Editorial Policy Checklist.

Statistics

For all statistical analyses, confirm that the following items are present in the figure legend, table legend, main text, or Methods section.

n/a	Confirmed	
☐	☒	The exact sample size (*n*) for each experimental group/condition, given as a discrete number and unit of measurement
☐	☒	A statement on whether measurements were taken from distinct samples or whether the same sample was measured repeatedly
☐	☒	The statistical test(s) used AND whether they are one- or two-sided<br>*Only common tests should be described solely by name; describe more complex techniques in the Methods section.*
☐	☒	A description of all covariates tested
☐	☒	A description of any assumptions or corrections, such as tests of normality and adjustment for multiple comparisons
☐	☒	A full description of the statistical parameters including central tendency (e.g. means) or other basic estimates (e.g. regression coefficient) AND variation (e.g. standard deviation) or associated estimates of uncertainty (e.g. confidence intervals)
☐	☒	For null hypothesis testing, the test statistic (e.g. *F*, *t*, *r*) with confidence intervals, effect sizes, degrees of freedom and *P* value noted<br>*Give P values as exact values whenever suitable.*
☒	☐	For Bayesian analysis, information on the choice of priors and Markov chain Monte Carlo settings
☒	☐	For hierarchical and complex designs, identification of the appropriate level for tests and full reporting of outcomes
☒	☐	Estimates of effect sizes (e.g. Cohen's *d*, Pearson's *r*), indicating how they were calculated

Our web collection on statistics for biologists contains articles on many of the points above.

Software and code

Policy information about availability of computer code

Data collection	Snapgene was used to visualize the mouse genome at the Pptc7 locus and design gRNA and primer sequences.<br>AMT Capture Engine software was used to capture electron micrographs.<br>QuantStudio Real Time PCR system software was used to capture qPCR data.<br>NIS Elements software was used to capture confocal flourescence images.<br>Image Studio sofware (LiCOR) was used to capture Western blot images.
Data analysis	Microsoft excel was used to analyze data, generate volcano plots, standard bar graphs, and to perform Student's t tests.<br>ImageJ was used to quantify mitochondrial area in Figures 3H and K, and to quantify band intensities in Figures 5 and S5.<br>BoxPlotR, a web-tool, was used to analyze data to generate boxplots in Figures 2A-E, 2H, 5P, S2G-I.<br>Coon OMSSA Proteomic Analysis Software Suite (COMPASS) was used to search RAW files for proteomic and phosphorproteomic data.

For manuscripts utilizing custom algorithms or software that are central to the research but not yet described in published literature, software must be made available to editors/reviewers. We strongly encourage code deposition in a community repository (e.g. GitHub). See the Nature Research guidelines for submitting code & software for further information.

Data

Policy information about availability of data

All manuscripts must include a data availability statement. This statement should provide the following information, where applicable:

- Accession codes, unique identifiers, or web links for publicly available datasets
- A list of figures that have associated raw data
- A description of any restrictions on data availability

The source data underlying Figures 2f-h, 5b, d, g, 6c-e, g-h and Supplementary Figures 2f-I, 3d, h, 5a, f, g, j, m, o, and 6a-d, g-I, k are provided as a Source Data file. The source data for proteomic and phosphoproteomic analyses are provided as Supplementary Datasets 1 and 2. The mass spectrometry proteomics data have

been deposited to the ProteomeXchange Consortium via the PRIDE partner repository with the dataset identifier PXD012743 [https://www.ebi.ac.uk/pride/archive/projects/PXD012743]. Other datasets generated during and/or analysed during the current study are available from the corresponding author on reasonable request.

Field-specific reporting

Please select the one below that is the best fit for your research. If you are not sure, read the appropriate sections before making your selection.

☒ Life sciences ☐ Behavioural & social sciences ☐ Ecological, evolutionary & environmental sciences

For a reference copy of the document with all sections, see nature.com/documents/nr-reporting-summary-flat.pdf

Life sciences study design

All studies must disclose on these points even when the disclosure is negative.

Sample size	Select biological measurements were taken for all mice studied (e.g. blood glucose, weight), and were included in the analysis. For other biological measurements (e.g. ketone measurements, lactate, acylcarnitine analysis, proteomics/phosphoproteomics), at least 5 biological replicates of wild type and Pptc7 knockout mice were used, which allowed a fold change of 0.5 to be calculated as significant ($p<0.05$; Type I error = 0.05; power 90%).
Data exclusions	In the acylcarnitine data analysis, 4 datapoints (with a datapoint defined as a single measurement in one acylcarnitine species from one biological replicate) were dropped due to being extreme outliers; outliers were tested identified using Grubbs' test via Graphpad software.
Replication	For physiological measurements (e.g. serum ketones), acylcarnitine analysis, metabolomic analysis, proteomic and phosphoproteomic analysis, mice were genotyped and chosen across multiple, independent litters for each experiment. For the biological follow up experiments (Figures 5 and 6), Tim50p mutant strain import assays (Figure 5) and import assays with HADH (Figure 6) were carried out by two independent laboratories (NMN in the Pagliarini lab and LM/FNV at the University of Frieberg) with similar reproducible results.
Randomization	Mice were sac'd at birth and randomly assigned a number. Tail tips were collected, genotyped, and the at least 5 mice from each genotype were randomly selected from this pool for each experiment, allowing randomization across litters (and likely sexes, as the sex of perinatal pups is indistinguishable at birth).
Blinding	Due to the randomized numbering system described above, data analysis was done blinded and deconvoluted by genotype after data were collected.

Reporting for specific materials, systems and methods

We require information from authors about some types of materials, experimental systems and methods used in many studies. Here, indicate whether each material, system or method listed is relevant to your study. If you are not sure if a list item applies to your research, read the appropriate section before selecting a response.

Materials & experimental systems

n/a	Involved in the study	
☐	☒	Antibodies
☐	☒	Eukaryotic cell lines
☒	☐	Palaeontology
☐	☒	Animals and other organisms
☒	☐	Human research participants
☒	☐	Clinical data

Methods

n/a	Involved in the study	
☒	☐	ChIP-seq
☒	☐	Flow cytometry
☒	☐	MRI-based neuroimaging

Antibodies

Antibodies used	anti-FLAG-M2 - Sigma #F1804<br>anti-VDAC - Abcam #ab18988<br>anti-OxPhos - Abcam #ab110412<br>anti-GRP75 - Abcam #ab2799<br>anti-PMPCB - developed and validated by Vogtle or Meisinger lab<br>anti-Tom70p - developed and validated by Vogtle or Meisinger lab<br>anti-Tom20p - developed and validated by Vogtle or Meisinger lab<br>anti-Por1p - Abcam #ab110326<br>anti-Tubulin - Abcam #ab59680
Validation	For FLAG: non-epitope expressing cell line used as negative control to ensure band specificity; various constructs used with different molecular weights, which were used for identification of the expected protein size.

For VDAC, OxPhos, Timm50, actin, GRP75, PMPCB, Por1p, tubulin - bands identified at expected molecular weights for protein products.

Eukaryotic cell lines

Policy information about cell lines

Cell line source(s)	293T cells were purchased from ATCC (catalog #CRL-3216), frozen at low passage, and only used to passage 20.
Authentication	Cell line was not authenticated.
Mycoplasma contamination	Cell line was not tested for mycoplasma contamination.
Commonly misidentified lines (See ICLAC register)	n/a

Animals and other organisms

Policy information about studies involving animals; ARRIVE guidelines recommended for reporting animal research

Laboratory animals	A Pptc7 knockout strain was generated using CRISPR-Cas9 technology in the C57BL/6J (B6) strain of Mus musculus [NCBItax:10090]. Mutational details are provided at MGI; Pptc7em1Pag at MGI:6094244, and Pptc7em2Pag at MGI:6143811.The Pptc7-/- Mus musculus strain was generated via CRISPR and is registered with MGI under the names C57BL/6J-Pptc7em1Pag (accession number MGI:6094249) and C57BL/6J-Pptc7em2Pag (accession number MGI:6143812); two strains are registered as two independently segregating Pptc7-/- alleles were generated in our founder mouse – one with a 4 bp deletion in exon 2 (hereby called E2; MGI:6094244), and one with a 1 bp deletion in exon 3 (hereby called E3; MGI:6143811). Wild type C57BL/6J mice were used for propagation of the strains as necessary.
Wild animals	The study did not involve wild animals.
Field-collected samples	The study did not include field-collected samples.
Ethics oversight	All animal work complied with ethical regulations for animal testing and research, and was done in accordance with IACUC approval by the College of Agricultural and Life Sciences (CALS) Animal Care and Use Committee at the University of Wisconsin-Madison (protocol/animal welfare assurance #A3368-01).

Note that full information on the approval of the study protocol must also be provided in the manuscript.
