# Supplementary material for: Pptc7 is an essential phosphatase for promoting mammalian mitochondrial metabolism and biogenesis
Source: Nat Commun. 2019 Jul 19;10:3197. doi: 10.1038/s41467-019-11047-6 (PMC6642090; doi:10.1038/s41467-019-11047-6)
Supplement: Supplementary file 2 — Peer Review File [file 41467_2019_11047_MOESM2_ESM.pdf]

Reviewers' comments:

Reviewer #1 (Remarks to the Author):

In the manuscript, the authors reported their studies on a mitochondrial phosphatase, Pptc7, and found that Pptc7-/- mice exhibited hypoketotic hypoglycemia, elevated acylcarnitines, and lactic acidosis. This gene is essential and Pptc7-/- mice died after birth. The diminished mitochondrial size was observed in the Pptc7-/- tissues. The proteome and phosphoproteome changes between the WT and Pptc7-/- tissues were quantified. Based on the phosphoproteome results, they further studied Timm50, a protein translocase complex subunit, as a putative Pptc7 substrate whose phosphorylation reduced import activity. In addition, they found that phosphorylation within or near the mitochondrial targeting sequences of several proteins could affect their import rates and matrix processing. This work is interesting and allows us to have a better understanding of the functions of this mitochondrial phosphatase, i.e. Pptc7.

It is uncertain whether this protein is only located in the mitochondria. It might be better to provide experimental evidence to demonstrate this.

In Figure 3A and B, for the non-mitochondrial proteome results, the authors stated that “These results showed no clear pattern of changes in non-mitochondrial proteins for either heart or liver.” Actually many proteins were regulated using the criteria of 1.5 fold. It might be better to perform more analysis for regulated proteins.

In Table 1, for Aco2 and Etfa, the ratios are 1.21, 1.27 and 1.29, and some P values are 1.000. Which criteria did they apply to list these proteins as the substrate candidates?

It might be better to share the raw files, and also make some supplemental tables about the proteome and phosphoproteome changes between the WT and Pptc7-/- tissues.

Reviewer #2 (Remarks to the Author):

This is, to my knowledge, the first murine knockdown of the relatively understudied apparent , relatively non-specific, phosphatase Pptc 7 from a leading group in discovering and characterizing mitochondria phosphatases. Regrettably the Pptc 7 knockout was perinatally lethal limiting the ability to interpret some of the data collected. The fact that this is a lethal defect is of interest alone, however, the cataclysmic events associated with neonatal death is confounding in the interpretation. Despite this limitation the authors have proposed, with reasonable but not complete data, that this phosphatase is part of the protein processing of the MTS sequence after import. This is a very interesting idea that would explain many of the observations made including decrease in mitochondrial content and broad impact on metabolic events. Indeed, this puts this phosphatase into a “house keeping” role explaining why it may not be associated with matrix kinase activity, very interesting. I encourage the authors to test his hypothesis more robustly by characterizing the phosphatase activity on different phosphorylated MTS sequence. I have several comments. My comments are in order of appearance in the paper and not in ranked priority of my concerns.
1) Page 4 “KO pups develop appropriately in utero”. This statement is not justified by detailed analysis, indeed later in on the same page “ weighed significantly less than their WT counterparts”. Very abnormal development can be sustained up to birth and the lower birth weight (a key indicator in neonatal survival) indicates that problems were initiated much earlier before birth. This statement should be omitted or validated with a fetus study. The lower birth weight suggests that defects even in the maternally supported animal was occurring and a singular focus on the birth transition might not be warranted.

2) The metabolomic and plasma metabolite studies were very consistent with a decrease in the ability to oxidize fatty acids throughout the body and was well done.
3) The data support the conclusion that the major metabolic effect is likely the loss of mitochondrial volume in, at least, heart and liver consistent with the inability to perform FAO. However, this condition would suggest that the tissue is already stressed as oxidative phosphorylation is necessary for normal mammalian development. Thus, studies on these pups will be confounded by the gross physiological and biochemical stress on the fetus and neonatal conditions. However, the conclusion is not unreasonable.
4) The authors extrapolate percent changes in phosphoprotein peptides (>1.5 fold) "suggests that it likely influences multiple processes within mitochondria." However, without establishing the occupancy of these phosphorylation events it is potentially misleading to over emphasis percent changes to actual impactful PTM on metabolic pathways. (in contrast to kinases and regulatory proteins were low occupancy can be impactful). This is highlighted by the assay presented in Figure 4 legend where the phosphor peptides had to be concentrated to be detected. I assume that all of the proteins detected with changes in protein phosphorylation were detected with the standard mass spectroscopy approaches, if the occupancy was significant why was phosphor-peptide enrichment required? This problem was illuminated in the introduction of the manuscript and needs to be addressed here.
5) The data concerning protein transport and processing in the matrix is very novel and generates a very compelling hypothesis that would be of great interest to the mitochondria community. It seems that phosphorylation events, that could even be non-specific, in the MTS might slow uptake but significantly impact MTS processing in the matrix. Thus protein gets in but cannot be used. If a phosphatase was responsible for this activity across a wide variety of imported proteins it might well be that the phosphatase is targeted to the MTS region of multiple proteins. Was this confirmed in your analysis of the increase in protein phosphorylation in the knockout primarily in the MTS region? Have you tested this phosphatase against phosphorylated MTS sequence (at different sites), this would go a long way in potentially having this phosphatase as a element in the processing chain of protein incorporation in the mitochondria.

Very minor comments
1) Having not followed the nomenclature developments in the mitochondria phosphatase field, in trying to follow the literature trail painted by the investigators it seems this phosphatase that was identified to be membrane bound in the inner membrane facing the matrix was called DSP-21. I have failed to find the connection between Pptc 7 or Pptc7p to DSP-21 but based on the nature of the reference provided I assume they are the same. One sentence clarifying the naming convention would be helpful.

Reviewer #3 (Remarks to the Author):

Mitochondrial protein biogenesis is regulated by phosphorylation reactions, but mechanisms and regulatory components are poorly studied. In this study, the authors elucidated the relevance of a previously non-characterized phosphatase of the mitochondrial matrix. They provide compelling evidence that this enzyme, Pptc7, is essential in mice, critical for the general production of mitochondria in heart and liver tissues, and more explicitly, for the accumulation of proteins of the mitochondrial matrix. By phospho-proteomics, the authors identify the essential inner membrane protein Timm50 as substrate of Pptc7. They suggest that the phosphorylation at threonine 33 of Timm50 prevents protein import, making Pptc7 a critical gate-opener for matrix import reactions. In addition, the authors identify a number of further substrates which are phosphorylated around their MPP processing site. Evidence from experiments with yeast mitochondria suggest that

dephosphorylation by Pptc7 is also relevant for the import and processing of these proteins.
Thus, the authors characterize a novel, essential component of the mitochondrial biogenesis system, identify several affected targets and come up with two complementary mechanisms by which Pptc7 exhibits a critical activating function during mitochondrial biogenesis. This is a highly exciting study which opens a door into a previously unknown aspect of cell biology which regulates critical reactions on different sites of the mitochondrial membranes. The quality is very high of this study and only a few minor aspects should be considered.

1
To understand the relevance of Timm50 phosphorylation, it will be essential to show data about how much of the Timm50 (in wildtype or ko mutant) is actually phosphorylated. Is this a major fraction as expected from the strong phenotype? Or a minor, but dominant-negative species? This needs to be experimentally addressed!

2
According to the phosphoproteome data presented in Fig. 4B-E, there is only a twofold difference in the degree of Timm50 phosphorylation between wt and ko mutant. This is surprising! Does this mean that other phosphatases overlap with Pptc7 which make this enzyme partially dispensable?

3
Figure 5M shows a very strong defect of protein import into yeast mitochondria that lack the yeast phosphatase Ptc7. It is not clear how comparable yeast and animals are. Nevertheless, this mutant could be interesting to test how important the Tim50 modification is for the general phenotype. The authors should show an experiment in which they express the Tim50-S104A mutant in this strain (in addition to the WT Tim50). If this rescues the import defect, the Ptc7-mediated dephosphorylation of Tim50 is presumably critical for the import defect of the Dptc7 mitochondria. If not, other Ptc7 substrates are presumably relevant. Both results are fully compatible with the model proposed in this paper.

4
Figure 5O shows a very superficial analysis of mitoCPR components. These factors are only moderately induced here (even though significantly). From the data shown, it remains unclear (or even unlikely) that this is a mitoCPR response. Since this is a superficial characterization of a yeast mutant, this aspect does anyway not fit well to the rest of the study. Figure 5O should therefore be removed and used for a more comprehensive yeast study in the future.

5
Figure 4E shows that VDAC1 is a Pptc7 substrate. Since VDAC1 is an outer membrane protein which presumably never explores the mitochondrial matrix, this observation is unexpected. The authors should comment on whether they expect a Pptc7-dependent phosphatase activity also in the intermembrane space.

6
The authors should cite the study on Pptc7 published by Gonzelez-Mariscal et al. (Biochim Biophys Acta Bioenerg. 2018 Nov;1859(11):1235-1248)

We thank the reviewers for their positive and constructive comments on our work, which we believe have helped us improve the manuscript. Please see below for a full point-by-point response to all reviewer comments.

Reviewers' comments:

Reviewer #1:

In the manuscript, the authors reported their studies on a mitochondrial phosphatase, Pptc7, and found that Pptc7-/- mice exhibited hypoketotic hypoglycemia, elevated acylcarnitines, and lactic acidosis. This gene is essential and Pptc7-/- mice died after birth. The diminished mitochondrial size was observed in the Pptc7-/- tissues. The proteome and phosphoproteome changes between the WT and Pptc7-/- tissues were quantified. Based on the phosphoproteome results, they further studied Timm50, a protein translocase complex subunit, as a putative Pptc7 substrate whose phosphorylation reduced import activity. In addition, they found that phosphorylation within or near the mitochondrial targeting sequences of several proteins could affect their import rates and matrix processing. This work is interesting and allows us to have a better understanding of the functions of this mitochondrial phosphatase, i.e. Pptc7.

It is uncertain whether this protein is only located in the mitochondria. It might be better to provide experimental evidence to demonstrate this.

We appreciate the opportunity to clarify this important point in our manuscript. There is significant literature precedence for the mitochondrial localization of Pptc7. First, this protein was identified in the MitoCarta study (Pagliarini et al. 2008, Cell), which was designed to identify resident (vs. "moonlighting") mitochondrial proteins. More recently, our lab demonstrated mitochondrial localization of a C-terminally FLAG-tagged Pptc7 construct in HEK293 cells via immunofluorescence (Floyd et al., Mol. Cell, 2016), with no other obvious extra-mitochondrial staining pattern. Pptc7 was further identified as a mitochondrial matrix protein using APEX-tagging (Rhee et al. Science, 2013), and was not found using this same strategy within the intramitochondrial membrane space (IMS) (Hung et al. Mol. Cell, 2014), suggesting (albeit, not proving) its sole matrix localization. Similar identification in matrix, but not in IMS, was found for Ptc7p in multiple independent studies that mapped the submitochondrial proteome (Vogtle et al. 2017 Nat Commun.; Morgenstern et al. 2017 Cell Rep., Vogtle et al. 2012 Mol Cell Proteomics). Collectively, we believe the reviewer will agree that these studies provide very strong evidence that Pptc7 is predominantly, if not exclusively, a mitochondrial matrix protein. This point and associated references have been added in the text (see Results under heading "Global knockout of Pptc7 causes perinatal lethality").

In Figure 3A and B, for the non-mitochondrial proteome results, the authors stated that "These results showed no clear pattern of changes in non-mitochondrial proteins for either heart or liver." Actually many proteins were regulated using the criteria of 1.5 fold. It might be better to perform more analysis for regulated proteins.

Indeed, this is a fair point, and we have updated our manuscript with new analyses. In our original text, we had meant to draw the parallel between the striking downregulation of the mitochondrial proteome versus the lack of a similar phenomenon in the non-mitochondrial proteome, but realize that our wording was unclear and potentially misleading. To address this, we have omitted the above statement from the text, and instead have replaced it with a statement regarding the suggested analysis.

To determine if there were pathways and processes dysregulated in the Pptc7 KO tissues, we filtered for all significantly upregulated or downregulated non-mitochondrial proteins found in our analysis (p-value <0.05). We then took the resulting lists of proteins and analyzed them for Gene Ontology (GO term) enrichment, and found that upregulated proteins across both tissues were associated with interesting signatures, including glutamine metabolism, RNA splicing, and nuclear transport. While these signatures are not nearly as strong as that for the mitochondrial effect, and it is not immediately apparent how these processes may be contributing to the phenotypes we see in the mouse, we have now included these analyses (Figure S2A and S2B) and have mentioned these observations in the text (see Results under heading "Loss of Pptc7 causes a post-transcriptional defect in mitochondrial biogenesis"). We thank the reviewer for this suggestion, which we believe has improved the manuscript.

In Table 1, for Aco2 and Etfa, the ratios are 1.21, 1.27 and 1.29, and some P values are 1.000. Which criteria did they apply to list these proteins as the substrate candidates?

We thank the reviewer for pointing out these inconsistencies in the table present in the first submission. The substrates in the table were selected because of their identification in both tissues in the KO mouse as well as their identification in either our previous Ptc7p yeast study (column *Δptc7*) and/or our previous phosphoproteomic study of a mouse model of type 2 diabetes (column MitoMod). While all substrates listed do reach statistical significance by p-value (all p-values <0.05), as pointed out by this reviewer, the multiple hypothesis corrected q-values are, for some reported substrates, not significant and can reach 1.00 in certain tissues, which we realize may lead to confusion as to why these proteins were selected. Therefore, we have eliminated the last segment of the table (which included the weakest candidate substrates, such as Aco2 and Etfa, mentioned by the reviewer) to make this table more transparent and easier to understand in terms of highlighting the top candidate Pptc7 substrates in both tissues (top portion of table), in liver only (middle portion of table) or in heart only (last portion of table).

It might be better to share the raw files, and also make some supplemental tables about the proteome and phosphoproteome changes between the WT and Pptc7-/- tissues.

The mass spectrometry proteomics data have been deposited to the ProteomeXchange Consortium via the PRIDE partner repository with the dataset identifier PXD012743. The data can be accessed by the reviewers at http://www.ebi.ac.uk/pride and accessed with the Username: reviewer08991@ebi.ac.uk and Password: hVKRL0Vn. Further, we have compiled two supplemental tables containing the total protein levels, quantified phosphopeptide levels, and the protein-normalized phosphopeptide levels (which correspond to the data shown in the manuscript) for both heart (Supplemental Table 1) and liver (Supplemental Table 2) tissues.

Reviewer #2 (Remarks to the Author):

This is, to my knowledge, the first murine knockdown of the relatively understudied apparent, relatively non-specific, phosphatase Pptc7 from a leading group in discovering and characterizing mitochondria phosphatases. Regrettably the Pptc7 knockout was perinatally lethal limiting the ability to interpret some of the data collected. The fact that this is a lethal defect is of interest alone, however, the cataclysmic events associated with neonatal death is confounding in the interpretation. Despite this limitation the authors have proposed, with reasonable but not complete data, that this phosphatase is part of the protein processing of the MTS sequence after import. This is a very interesting idea that would explain many of the observations made including decrease in mitochondrial content and broad impact on metabolic events. Indeed, this puts this

phosphatase into a "house keeping" role explaining why it may not be associated with matrix kinase activity, very interesting. I encourage the authors to test his hypothesis more robustly by characterizing the phosphatase activity on different phosphorylated MTS sequence. I have several comments. My comments are in order of appearance in the paper and not in ranked priority of my concerns.

1) Page 4 "KO pups develop appropriately in utero". This statement is not justified by detailed analysis, indeed later in on the same page " weighed significantly less than their WT counterparts". Very abnormal development can be sustained up to birth and the lower birth weight (a key indicator in neonatal survival) indicates that problems were initiated much earlier before birth. This statement should be omitted or validated with a fetus study. The lower birth weight suggests that defects even in the maternally supported animal was occurring and a singular focus on the birth transition might not be warranted.

We thank the reviewer for bringing this issue to our attention. We had intended this statement to mean that the KO pups maintain viability *in utero*, but the wording here was overreaching, and has been corrected. We have omitted the first statement from the text, and amended the text to point out the observations put forth by the reviewer by calling attention to the low birth weight as well as the decreased embryo size, and how both of these observations likely reflect metabolic deficiencies in utero and after birth (See "Pptc7-null mice have defects associated with inborn errors of metabolism" for text changes).

2) The metabolomic and plasma metabolite studies were very consistent with a decrease in the ability to oxidize fatty acids throughout the body and was well done.

We thank the reviewer for this feedback.

3) The data support the conclusion that the major metabolic effect is likely the loss of mitochondrial volume in, at least, heart and liver consistent with the inability to perform FAO. However, this condition would suggest that the tissue is already stressed as oxidative phosphorylation is necessary for normal mammalian development. Thus, studies on these pups will be confounded by the gross physiological and biochemical stress on the fetus and neonatal conditions. However, the conclusion is not unreasonable.

We agree with the reviewer's conclusion, and acknowledge that there is much work left to be done to establish the role of Pptc7 in the absence of the potentially confounding factors identified here. We have begun establishing an inducible KO mouse that hopefully will enable us to study the role of Pptc7 in older mice following normal development in the future. However, the timeline of generating these mice is not consistent with this revision.

4) The authors extrapolate percent changes in phosphoprotein peptides (>1.5 fold) "suggests that it likely influences multiple processes within mitochondria." However, without establishing the occupancy of these phosphorylation events it is potentially misleading to over emphasis percent changes to actual impactful PTM on metabolic pathways. (in contrast to kinases and regulatory proteins were low occupancy can be impactful). This is highlighted by the assay presented in Figure 4 legend where the phosphor peptides had to be concentrated to be detected. I assume that all of the proteins detected with changes in protein phosphorylation were detected with the standard mass spectroscopy approaches, if the occupancy was significant why was phosphor-peptide enrichment required? This problem was illuminated in the introduction of the manuscript and needs to be addressed here.

We acknowledge that this is an important point, and we appreciate the reviewer's comments on our approach with respect to phosphopeptide enrichment. While we agree that substantially occupied phosphorylation events may be detectable by mass spectrometry without enrichment, use of an enrichment step remains standard practice for us and others for two reasons. The first, consistent with the reviewer's comment, is that many phosphorylation events are present at low stoichiometry, and that enrichment greatly increases the number of potentially relevant phosphorylation events identified (even if those are present at low stoichiometry). The second reason is that the phosphopeptide signal can be suppressed during ionization, necessitating a higher input to retain the signal throughout the duration of the experiment. A final reason that enrichment was necessary for this particular experiment is that we used single tissues from newborn mouse pups, which were highly limiting in total input mass.

We concede that we cannot distinguish between high and low occupancy modifications using the TMT proteomics methodology. Absolute stoichiometries could be calculated by synthesizing custom phosphorylated and non-phosphorylated peptides and establishing standard curves via a targeted mass spectrometry analsysis. While possible, we hesitated to pursue this time- and cost-intensive approach for this revision for a few reasons. First, without yet knowing the mechanism by which phosphorylation is affecting function, it is difficult to know what the percent occupancy would mean biologically. Second, our data suggest that phosphorylation could cause protein instability in some instances (e.g. Iscu S15E expression, Figure S6I), thus leading to an underestimation of phospho-levels, even if we had the requisite reagents. Third, our recent work (Guo et al., JCB 2017) revealed that, even for the well-characterized PDC complex, the mass spectrometry-based phosphoproteomics analyses markedly underestimated the phosphorylation levels observed by Western blots (using PDC phospho-specific antibodies). Finally, work by the Gygi group (Wu et al., Nature Methods 2011) concluded that "*analysis of 25 fungal species … suggests that stoichiometry does not positively correlate with a site's biological essentialness."* As we have discussed in the past, this is similar to what is observed with acylation and sirtuins. It appears now that acylation stoichiometry is most often extremely low and generated non-enzymatically; however, mice lacking, for instance, *Sirt3* have, when stressed, exhibited a notable metabolic phenotype. Nonetheless, we have changed the wording in the statement cited by the reviewer to "suggests it may influence multiple processes within the mitochondria" to avoid the potential overemphasis on meaningful, regulatory modifications, and intend to establish precise stoichiometry data in future work.

5) The data concerning protein transport and processing in the matrix is very novel and generates a very compelling hypothesis that would be of great interest to the mitochondria community. It seems that phosphorylation events, that could even be non-specific, in the MTS might slow uptake but significantly impact MTS processing in the matrix. Thus protein gets in but cannot be used. If a phosphatase was responsible for this activity across a wide variety of imported proteins it might well be that the phosphatase is targeted to the MTS region of multiple proteins. Was this confirmed in your analysis of the increase in protein phosphorylation in the knockout primarily in the MTS region? Have you tested this phosphatase against phosphorylated MTS sequence (at different sites), this would go a long way in potentially having this phosphatase as a element in the processing chain of protein incorporation in the mitochondria.

We thank the reviewer for their appreciation of the model we have put forth in the manuscript. To address the questions asked by the reviewer, we would first point to current data in the manuscript demonstrating 1) that Pptc7 can directly phosphorylate both of the phospho-MTS substrates we tested (of five total identified that are present in both Pptc7 KO tissues) (HADH, Figure 6C) and Ethe1 (Figure S6A), and 2) that phosphomimetic mutation of four of these (HADH, Ethe1, NAXE, and Iscu) is sufficient to cause processing defects or diminish protein stability (Figures 6D, S6G-

I). To add to the model, we now have further demonstrated that catalytically active PPTC7, but not its inactive counterpart (D78A), can directly dephosphorylate NAXE pS43 (Figure S6B), Pdk1 pS11 (Figure S6C), and Iscu (Figure S6D). Notably, the dephosphorylation for Pdk1 pS11 is not very efficient, but this may be because the phosphosubstrate we found was dually phosphorylated at T11 and S12, or because we had to mutate the threonine to a phosphoserine due to the limitations of our phosphoincorporation system. Despite these limitations, it seems as though Pptc7 can at least partially if not fully dephosphorylate all five tested phospho-MTS substrates directly in vitro, suggesting that these may be bona fide substrates for the phosphatase in vivo.

Very minor comments

1) Having not followed the nomenclature developments in the mitochondria phosphatase field, in trying to follow the literature trail painted by the investigators it seems this phosphatase that was identified to be membrane bound in the inner membrane facing the matrix was called DSP-21. I have failed to find the connection between Pptc 7 or Pptc7p to DSP-21 but based on the nature of the reference provided I assume they are the same. One sentence clarifying the naming convention would be helpful.

DSP-21 is not the same phosphatase as Pptc7; Pptc7 is a PP2C family phosphatase that uses a distinct catalytic mechanism involving divalent cations, whereas DSP-21 is a dual specificity phosphatase that functions via a canonical protein tyrosine phosphatase catalytic motif (HCX_5R). To address this in the text, we have added a statement identifying Pptc7 as a PP2C phosphatase (See "*Global knockout of Pptc7 causes perinatal lethality*").

Reviewer #3 (Remarks to the Author):

Mitochondrial protein biogenesis is regulated by phosphorylation reactions, but mechanisms and regulatory components are poorly studied. In this study, the authors elucidated the relevance of a previously non-characterized phosphatase of the mitochondrial matrix. They provide compelling evidence that this enzyme, Pptc7, is essential in mice, critical for the general production of mitochondria in heart and liver tissues, and more explicitly, for the accumulation of proteins of the mitochondrial matrix. By phospho-proteomics, the authors identify the essential inner membrane protein Timm50 as substrate of Pptc7. They suggest that the phosphorylation at threonine 33 of Timm50 prevents protein import, making Pptc7 a critical gate-opener for matrix import reactions. In addition, the authors identify a number of further substrates which are phosphorylated around their MPP processing site. Evidence from experiments with yeast mitochondria suggest that dephosphorylation by Pptc7 is also relevant for the import and processing of these proteins. Thus, the authors characterize a novel, essential component of the mitochondrial biogenesis system, identify several affected targets and come up with two complementary mechanisms by which Pptc7 exhibits a critical activating function during mitochondrial biogenesis. This is a highly exciting study which opens a door into a previously unknown aspect of cell biology which regulates critical reactions on different sites of the mitochondrial membranes. The quality is very high of this study and only a few minor aspects should be considered.

We thank the reviewer for the overall positive review of our work.

1. To understand the relevance of Timm50 phosphorylation, it will be essential to show data about how much of the Timm50 (in wildtype or ko mutant) is actually phosphorylated. Is this a major fraction as expected from the strong phenotype? Or a minor, but dominant-negative species? This needs to be experimentally addressed!

As noted above in response to Reviewer #2, absolute stoichiometries could be calculated by synthesizing custom phosphorylated and non-phosphorylated peptides and establishing standard curves via mass spectrometry. While possible, we hesitated to pursue this time- and cost-intensive approach for this revision for a few reasons. First, without yet knowing the mechanism by which phosphorylation is affecting function, it is difficult to know what percent occupancy would mean biologically. Second, the finding that the phosphomimetic Timm50 T33E decreases protein abundance suggests that phosphorylation could cause protein instability, thus leading to an underestimation of phospho-levels, even if we had the requisite reagents. Third, our recent work (Guo et al., JCB 2017) revealed that, even for the well-characterized PDC complex, the phosphoproteomics analyses markedly underestimated the phosphorylation levels observed by Western blots (using PDC phospho-specific antibodies). Finally, work by the Gygi group (Wu et al., Nature Methods 2011) concluded that "*analysis of 25 fungal species … suggests that stoichiometry does not positively correlate with a site's biological essentialness."* As we have discussed in the past, this is similar what is observed with acylation and sirtuins. It appears now that acylation stoichiometry is most often extremely low and generated non-enzymatically; however, mice lacking, for instance, *Sirt3* have, when stressed, have a notable metabolic phenotype. Nonetheless, we have changed the wording in the above statement to "suggests it may influence multiple processes within the mitochondria" to avoid the potential overemphasis on meaningful, regulatory modifications, as suggested by the reviewer.

We should note that we also attempted to estimate Timm50 stoichiometry using the PhosTag system. These experiments were complicated by the low Timm50 levels in *Pptc7* KO tissues, making it difficult to discern band shifts that could correspond to phosphorylated Timm50. Unfortunately, but interestingly, phosphorylation of Timm50 in mice may promote protein turnover. We generated a FLAG-tagged Timm50 construct and generated non-phosphorylatable (T33A) and phosphomimetic (T33E) mutants, and found that either mutation at T33 markedly reduced protein expression, with the phosphomimetic mutant virtually undetectable relative to the other two species (see rebuttal Figure 1 below). Collectively, these data lead us to propose that phosphorylation of Timm50 at T33 destabilizes the protein, thereby leading to an underestimation of phosphorylation fold changes and making visualization via PhosTag difficult. We cannot, however, dismiss the fact that we may be unable to visualize the phosphorylated species due to protein-specific technical issues, or because the Timm50 T33 phosphorylation is low abundance and thus is not detected as a band shift. We intend to establish precise stoichiometry data using the mass spectrometry method noted above in future work.

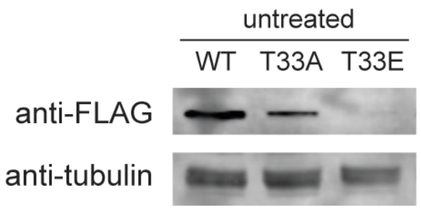

Figure 1: Timm50 phosphomimetic T33E mutant is destabilized. C-terminally FLAG-tagged Timm50 constructs encoding wild type (WT), non-phosphorylatable (T33A) or phosphomimetic (T33E) Timm50 were expressed 293 cells and analyzed by Western blotting (anti-FLAG for Timm50 isoforms, tubulin as a loading control).

2. According to the phosphoproteome data presented in Fig. 4B-E, there is only a twofold difference in the degree of Timm50 phosphorylation between wt and ko mutant. This is surprising! Does this mean that other phosphatases overlap with Pptc7 which make this enzyme partially dispensable?

We appreciate the opportunity to comment on this interesting and important point. First, it is possible that the relative two-fold change in signal between the WT and KO samples is an underestimate due to technical limitations of the TMT proteomics approach. It is well accepted

that the relative differences in fold change in multiplexed tagging proteomics experiments, such as that used in this manuscript, are commonly associated with dynamic range compression. This occurs because near isobaric ions are co-isolated with the target ions, and can also interfere with fragmentation, which skews reporter ion intensities. This interference typically causes an underestimation of the true fold changes between samples, and we believe that this likely contributes to the lower than expected difference in quantification between WT and KO samples. As noted above, this was clear in our recent evaluation of the Ptc6p-based dephosphorylation of the yeast pyruvate dehydrogenase complex (Guo et al., JBC 2017). It is worth noting that the Timm50 phosphorylation event is near the top of quantified phosphoisoforms changing in both heart and liver tissues, demonstrating it is amongst the phosphoisoforms with the highest fold change relative to the thousands of other quantified phosphopeptides. Second, as noted above, phosphorylation seems to cause Timm50 destabilization. It is possible that we are blind to the full population of phosphorylated Timm50 because it is turned over, leading to the overall loss of Timm50 protein content.

Overall, we of course cannot be certain that Pptc7 is uniquely responsible for the dephosphoryation of each discovered substrate. However, we would argue, similar to the reviewer, that at best other phosphatases could be partially redundant, as substantial redundancy would protect against the extreme phenotypes of the KO mouse, and dampen the increase of so many phosphosites. Thus, while it is reasonable to propose that other phosphatases partially compensate for Pptc7 loss, potentially reducing the overall phosphorylation fold-changes, the biological effects lead us to favor Pptc7 serving as the main driver of dephosphorylation for most of these putative substrates in vivo.

3. Figure 5M shows a very strong defect of protein import into yeast mitochondria that lack the yeast phosphatase Ptc7. It is not clear how comparable yeast and animals are. Nevertheless, this mutant could be interesting to test how important the Tim50 modification is for the general phenotype. The authors should show an experiment in which they express the Tim50-S104A mutant in this strain (in addition to the WT Tim50). If this rescues the import defect, the Ptc7-mediated dephosphorylation of Tim50 is presumably critical for the import defect of the Dptc7 mitochondria. If not, other Ptc7 substrates are presumably relevant. Both results are fully compatible with the model proposed in this paper.

We thank the reviewer for this suggestion. We have now performed this experiment, in which we overexpressed the Tim50 S104A mutant in the Δ*ptc7* W303 strain that has substantially decreased mitochondrial import (Figures 5G and S5O). As predicted by the reviewer, the overexpression of Tim50 S104A was sufficient to partially, but not completely, rescue the import defect in the Δ*ptc7* yeast, suggesting that phosphorylation at Tim50 S104 is relevant to the import defects seen in Δ*ptc7* yeast, but that other factors likely also contribute. This is not surprising, as multiple other import components have been previously identified as candidate Ptc7p substrates (Guo et al., JBC 2017), and we have previously established that metabolic deficiencies occur in the Δ*ptc7* strain (Guo et al. Cell Rep 2017), suggesting non-specific mitochondrial deficiencies, such as decreased membrane potential, may also contribute to this phenotype. These new data showing the S104A partial rescue are now shown in the main text (Figures 5G-I), and are commented upon in the text (See “Pptc7-mediated modulation of Timm50 function is conserved through S. cerevisiae”).

4. Figure 5O shows a very superficial analysis of mitoCPR components. These factors are only moderately induced here (even though significantly). From the data shown, it remains unclear (or even unlikely) that this is a mitoCPR response. Since this is a superficial characterization of a

yeast mutant, this aspect does anyway not fit well to the rest of the study. Figure 5O should therefore be removed and used for a more comprehensive yeast study in the future.

We have eliminated this portion of Figure 5, along with the text.

5. Figure 4E shows that VDAC1 is a Pptc7 substrate. Since VDAC1 is an outer membrane protein which presumably never explores the mitochondrial matrix, this observation is unexpected. The authors should comment on whether they expect a Pptc7-dependent phosphatase activity also in the intermembrane space.

This is an important point, and we thank the reviewer for making this observation. Reviewer #1 also commented about the localization of Pptc7, and we have added text and citations of literature that support matrix localization of this phosphatase to the manuscript (see Results under heading "Global knockout of Pptc7 causes perinatal lethality"). While we cannot rule out a mechanism for Pptc7 activity outside of the mitochondrion, there is no current evidence to support this model, and we predict that the increase in phosphorylation on Vdac1 is likely a downstream consequence of Pptc7 ablation, and we have stated this in the text (see Results under heading "Phosphoproteomic analysis of Pptc7-/- mice reveals candidate substrates"). Indeed, many non-mitochondrial phosphoisoforms were also identified as statistically significant in their alterations, and we also identify phosphosites that decrease in response to the loss of the phosphatase in both tissues. These data suggest that the signaling network is unsurprisingly complex, and teasing out initial perturbations due to Pptc7 loss, versus those that may be downstream, will be a key challenge in following up this work. Interestingly, Vdac1 has recently been implicated in mitochondrial protein import (Ellenrieder et al. Mol Cell 2019), suggesting that this may be a compensatory response to the import defect caused by Pptc7 loss.

6. The authors should cite the study on Pptc7 published by Gonzelez-Mariscal et al. (Biochim Biophys Acta Bioenerg. 2018 Nov;1859(11):1235-1248).

We have included this citation (reference 21) in our manuscript.

REVIEWERS' COMMENTS:

Reviewer #1 (Remarks to the Author):

The changes and responses are satisfactory.

Reviewer #2 (Remarks to the Author):

I think the manuscript has improved with this review. Specifically the further demonstration that this phosphatase can act on multiple sites support its house keeping role. Note: "demonstrated that catalytically active PPTC7, but not its inactive counterpart (D78A), can directly dephosphorylate NAXE pS43 (Figure S6B), Pdk1pS11 (Figure S6C), and Iscu (Figure S6D).

I still believe that the authors are over emphasizing the role of protein phosphorylation. The reference to the comparison of physical methods (mass spec) with affinity measures (antibodies) is weak, while the comparison to the fungal data is also confusing as it is unclear whether the post-translational events are related to the phenotype. In any event, what this study does show is that these phosphatase may remove the phosphorylation decorations that occur at a very low level in the transfer to the mitochondria and likely is in place to preserve function of these systems. This is an important function and should not be confused with signaling...... I leave any changes up to the authors and editors.

Reviewer #3 (Remarks to the Author):

The authors addressed satisfactorily all points raised on the initial version of this study. I support publication of this ms in its present form.
